# Alumina-enriched sunflower bio-oil in machining of Hastelloy C-276: a fuzzy Mamdani model-aided sustainable manufacturing paradigm

**DOI:** 10.1038/s41598-024-80254-z

**Published:** 2024-11-25

**Authors:** Binayak Sen, Abhijit Bhowmik, Gurbhej Singh, Vishwesh Mishra, Shantanu Debnath, Rustem Zairov, Muhammad Imam Ammarullah

**Affiliations:** 1 Centre for Computational Modeling, Chennai Institute of Technology, Chennai, 600069 Tamil Nadu India; 2 Department of Mechanical Engineering, Chennai Institute of Technology, Chennai, 600069 Tamil Nadu India; 3 Department of Mechanical Engineering, Dream Institute of Technology, Kolkata, 700104 West Bengal India; 4 Centre for Research Impact and Outcome, Chitkara University Institute of Engineering and Technology, Chitkara University, Rajpura, 140401 Punjab India; 5 Department of Mechanical Engineering, Amritsar Group of Colleges, Amritsar, 143109 Punjab India; 6 Chitkara Centre for Research and Development, Chitkara University, Baddi, 174103 Himachal Pradesh India; 7 Department of Mechanical Engineering, Institute of Engineering and Technology, GLA University, Mathura, 281406 Uttar Pradesh India; 8 Department of Mechanical Engineering, SRKR Engineering College, Bhimavaram, 534204 Andhrapradesh India; 9 Aleksander Butlerov Institute of Chemistry, Kazan Federal University, 1/29 Lobachevskogo Str., 420008 Kazan, Russian Federation; 10 Department of Mechanical Engineering, Faculty of Engineering, Universitas Diponegoro, Semarang, 50275 Central Java Indonesia; 11 Undip Biomechanics Engineering & Research Centre (UBM-ERC), Universitas Diponegoro, Semarang, 50275 Central Java Indonesia

**Keywords:** Alumina-enriched sunflower bio-oil, Minimum quantity lubrication (MQL), Tool wear mechanism, Surface protection film, Fuzzy Mamdani model, Sustainability index, Engineering, Mechanical engineering

## Abstract

With the increasing emphasis on sustainable manufacturing practices, eco-friendly lubricants have gained significant attention to moderate the friction coefficient at the tool-work interface. In line with this, the contemporary study aimed to examine the viability of Alumina-enriched sunflower bio-oil as a metalworking fluid. Different volume fractions of Alumina nanoparticles (varying from 0 to 1 vol%) were mixed with sunflower bio-oil, and the physical properties, for instance, contact angle and dynamic viscosity, were analyzed to determine the optimal concentration of Alumina. Subsequently, machining experiments were executed on Hastelloy C-276 under various lubricating conditions, including dry cutting, compressed air, sunflower bio-oil, and 0.6 vol% Alumina-sunflower bio-oil. A comparative analysis among these lubricating mediums demonstrated that sunflower bio-oil with a 0.6 vol% Alumina concentration outperformed others, resulting in a significant reduction of surface roughness, and tool wear by 73.31%, and 82.14% respectively when compared to dry machining. Besides, the utilization of 0.6 vol% Alumina-sunflower bio-oil has demonstrated a reduction of 17.86% in total machining cost, along with reductions of 15.44% in energy consumption and carbon emissions, when compared to dry machining. Finally, a Taguchi-designed experiment consisting of sixteen trials was performed in different lubricating conditions, and a Fuzzy-Mamdani model was employed to achieve a sustainable machining environment. The sustainability assessment results indicated that a cutting speed of 75 m/min, feed of 0.05 mm/tooth, depth of cut of 0.15 mm, and the utilization of the 0.6 vol% Alumina-sunflower bio-oil resulted in the most sustainable machining environment, with the highest Multi-Performance Characteristics Index of 0.75.

## Introduction

**Fig. 1:**
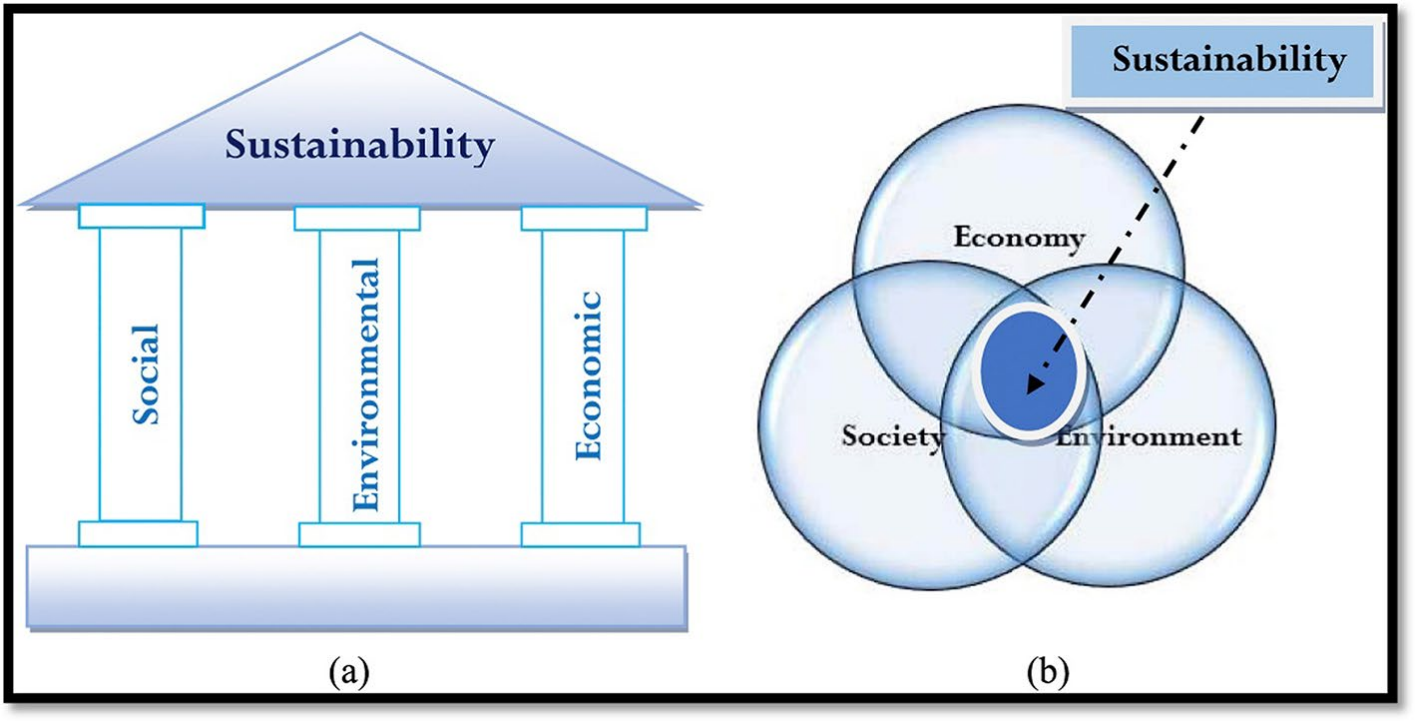
Three fundamental pillars of sustainability^4^.

Nickel-based superalloys are famous among engineering sectors for their enormous aptitude to maintain mechanical assets in extreme conditions. Consequently, these alloys are frequently applied in manufacturing fields, including turbine, aircraft, and maritime^1–2^. Though these alloys are extensively utilized, their machinability must be improved. Modern-time scientists have suggested various techniques to advance its machinability. However, the most suitable way is the employment of flood lubrication technology during machining^3–4^. Over the last decade, a concept like green and sustainable manufacturing has become an area of exploration. Thus, most manufacturing industries are eyeing a lubrication system that fulfills the directives of ISO 14,000^5–6^. Therefore, the minimum quantity lubrication (MQL) method has gained popularity, strengthening three fundamental pillars of sustainability (Fig. 1).

Because polar groups are present, vegetable oils are supposed to be strong contenders as MQL fluid^7^. On the other hand, some literature has shown that vegetable oils are auto-oxidants, meaning they tend to react with oxygen in the air, leading to oxidation. This auto-oxidation process can weaken the chemical structure over time. Most polar bonds were, therefore, disrupted at high temperatures^8^. Nevertheless, modern studies have revealed that nanoparticles can enhance the lubricity of neat vegetable oil^9^. Hence, assessing the lubricating ability of nanoparticle mixed vegetable oils is of current research interest. Initially, Zhang et al.^10^ scrutinized the lubricating performance of various nano-lubricants. As a result, MoS_2_ particles were mixed with sunflower, rapeseed, and soybean oils. They discovered that nanoparticles could enhance the tribological characteristics of pure vegetable oils. Additionally, the researchers noted that prepared lubricants provide a smoother grounded surface compared to other mediums. Later, Jia et al.^11^ discovered that castor and soybean oil with MoS_2_ doping agents possesses superior machining properties. Researchers observed the variation in surface roughness, specific grinding energy, and tangential cutting force in machining to evaluate the potential of using nanoparticle-mixed vegetable oils as a lubricant. The research outcomes indicate that lubricant enhanced with MoS_2_ nanoparticles performed better than conventional lubricating medium. Singh et al.^12^ explored the lubricity of graphene-mixed vegetable oil in Titanium alloy grinding. The research reported that a lubricating coating was created on the ground surface with nanoparticle-enhanced vegetable oil, which showed excellent machining performance. Gajrani et al.^13^ inspected the lubricity of nano-doped vegetable oil-based lubricants during hard machining operations. By examining their thermo-physical attributes, the authors found the best proportion of nanoparticles. Then, a detailed machining analysis was conducted in mineral oil, vegetable oil, CaF2 nanofluid, and MoS2 nanofluid-based lubricating medium. The findings of this practice have shown that vegetable oil with 0.3% MoS_2_ behaves better than other mediums. Mixing nanoparticles in vegetable oil has shown encouraging results in enhancing machinability, sustainability, and productivity. In another holistic work of Singh et al.^14^, various nanoparticles, i.e., graphene, graphite, and molybdenum disulfide, were mixed with canola oil to grind the aluminium-titanium-based alloy. The findings of this specific investigation unequivocally demonstrate that the utilization of graphene-mixed canola oil confers a notable enhancement upon the machining process, chiefly manifesting in reduced surface roughness, diminished cutting forces, and a simultaneous reduction in specific grinding energy consumption. Pal et al.^15^ rigorously conducted empirical investigations into the lubricating efficacy of graphene-mixed vegetable oil during the machining of AISI 321 steel. The researchers suggest that the incorporation of graphene powders into vegetable oil has substantiated its inherent potential to effectually diminish cutting forces, torque requirements, surface roughness, and coefficients of friction. Sirin et al.^16^ employed coated ceramic tools to turn a nickel-based alloy, subjecting it to dry and MQL-based conditions. The output parameters, like the roughness of the turned surface, tool-chip temperature, wear morphology of SiAlON ceramic tool, micro-hardness, and cutting force fluctuation, were investigated in detail. The results claim that the minimum quantity of nanofluids performed better than other conditions. Öndin et al.^17^ also used MWCNTs-mixed nanofluids to machinate martensitic steel. All machining trials were meticulously conducted under three distinct conditions: dry machining, MQL, and nano-MQL. The ensuing discussions encompassed an in-depth analysis of surface topography, cutting temperatures, and underlying tool wear mechanisms. The authors reveal that better machining performances could be obtained with a minimum quantity of nanofluids. Maruda et al.^18^ discuss the development of eco-friendly cooling methods for hard-to-cut materials, such as titanium alloy Ti6Al4V. Various nanofluids are used during cutting to minimize friction and heat accumulation at the tool edge. This paper analyzes tool wear indicators and mechanisms after turning with different cooling techniques: MQL with copper nanoparticles (CuNPs), dry machining, and MQL without nanoparticles. Four CuNP sizes (22 nm, 35 nm, 65 nm, and 80 nm) were tested. SEM analysis revealed that smaller CuNPs reduced flank wear and crater depth on the rake face. Specifically, using MQL with 22 nm CuNPs resulted in tool wear reductions of 25.7–55% compared to dry machining and 11.7–39.2% compared to MQL without nanoparticles. However, nanofluids, while enhancing cooling and lubrication in machining, pose environmental and human toxicity risks. Nanoparticles can penetrate biological tissues, causing respiratory, cardiovascular, and cellular damage in humans, especially when inhaled or ingested during machining operations. Additionally, improper disposal of nanofluids contaminates soil and water, impacting ecosystems and harming aquatic life^2^. The persistence and bioaccumulation of nanoparticles in the environment underscore the need for safer handling, disposal methods, and alternative eco-friendly cooling solutions in machining processes.

In recent years, electrostatic minimum quantity lubrication (EMQL) has emerged as a sustainable machining technique that significantly enhances surface quality and integrity^19–20^. This method builds on traditional minimum quantity lubrication by utilizing high voltage to negatively charge the oil and compressed air while positively charging the workpiece, leading to improved lubrication and cutting efficiency^21^. Research by Lv et al.^22^ demonstrated that nanoparticle-enhanced EMQL (NEMQL) significantly improved tribological conditions and reduced friction when machining AISI 1040, achieving tool wear reductions of 53.1%, 40%, and 25% compared to oil-based MQL, oil-based EMQL, and nanofluid MQL, respectively. Furthermore, NEMQL exhibited better performance with lower oil mist concentrations and a reduced coefficient of friction. Huang et al.^23^ identified optimal conditions for EMQL at 0.3 MPa air pressure and 7 kV voltage, attributing enhanced performance to a lubricating layer formed by an oxide layer and adsorption film. Meanwhile, Khanna et al.^24^ emphasized the need for precise machining of Ti6Al4V ELI implants, crucial for high-load medical applications, highlighting the challenges posed by titanium’s machining properties. This study explores sustainable techniques, including EMQL and liquid carbon dioxide (LCO2), focusing on hole quality, wear mechanisms, and chip morphology. The results indicated that while EMQL produced rougher surfaces (42–71% higher Rz values), LCO2 significantly improved tool life (416% enhancement). Additionally, hybrid nanoparticles in EMQL (HNEMQL) demonstrated reduced power consumption while enhancing surface finish and chip formation in machining 15–5 PHSS. This study also investigates drilling VT-20 using various methods, with HNPEMQL employing hybrid nanofluids proving most effective in improving drilling performance and reducing tool wear by 102%. However, EMQL technology presents several disadvantages from environmental and sustainability perspectives. While it reduces coolant consumption, the process often relies on synthetic lubricants, which may contain harmful chemicals that pose risks to soil and water ecosystems, undermining the overall goal of sustainable manufacturing practices^25–26^.

The utilization of a minimal quantity of nanofluids has drawn attention on a global scale. To optimize the efficacy of MQL technology, it is crucial to identify and explore emerging lubricants. According to reports, sunflower bio-oil has specific qualities that make it a good lubricant. Sunflower bio-oil has higher heat tolerance and oxidation resistance due to its high saturated fat content^27–28^. Furthermore, based on the previous literature, it has been revealed that Alumina nanoparticles can create surface protection layers and control heat build-up by reducing friction in machining^29–30^. Thus, the impact of Alumina-enhanced sunflower bio-oil on the machinability of Hastelloy C-276 was thoroughly investigated in this study by measuring various machining indices, including surface roughness, and tool wear. Moreover, the calculation of parameters, including machining cost, energy consumption, and carbon emissions, was performed for each lubricating medium. Finally, the Fuzzy-Mamdani model has been used to investigate sustainability based on a comparative evaluation of dry, compressed air, sunflower bio-oil, and Alumina-sunflower bio-oil mediums. For each experimental run, the multi-performance characteristics index (MPCI) value was determined using the triangle fuzzy membership function, and a maximum MPCI value denotes a machining environment that is the most sustainable. The following is a concise summary of the study’s main contributions:

- Synthesis of Alumina-enriched sunflower bio-oil based lubricant and investigation of its physical properties such as contact angle and dynamic viscosity.
- Examination of the surface topology, and tool wear mechanism for determining the lubricating potential of the in-house developed lubricant.
- Investigating the impact of various lubricating mediums on total machining cost, energy consumption, and carbon footprint in the machining of Hastelloy C-276.
- Sustainability evaluation of the machining process with the help of the Fuzzy-Mamdani model concerning the technical, economic, and ecological aspects.

## Materials and methods

### Preparation of nano-lubricants

**Fig. 2:**
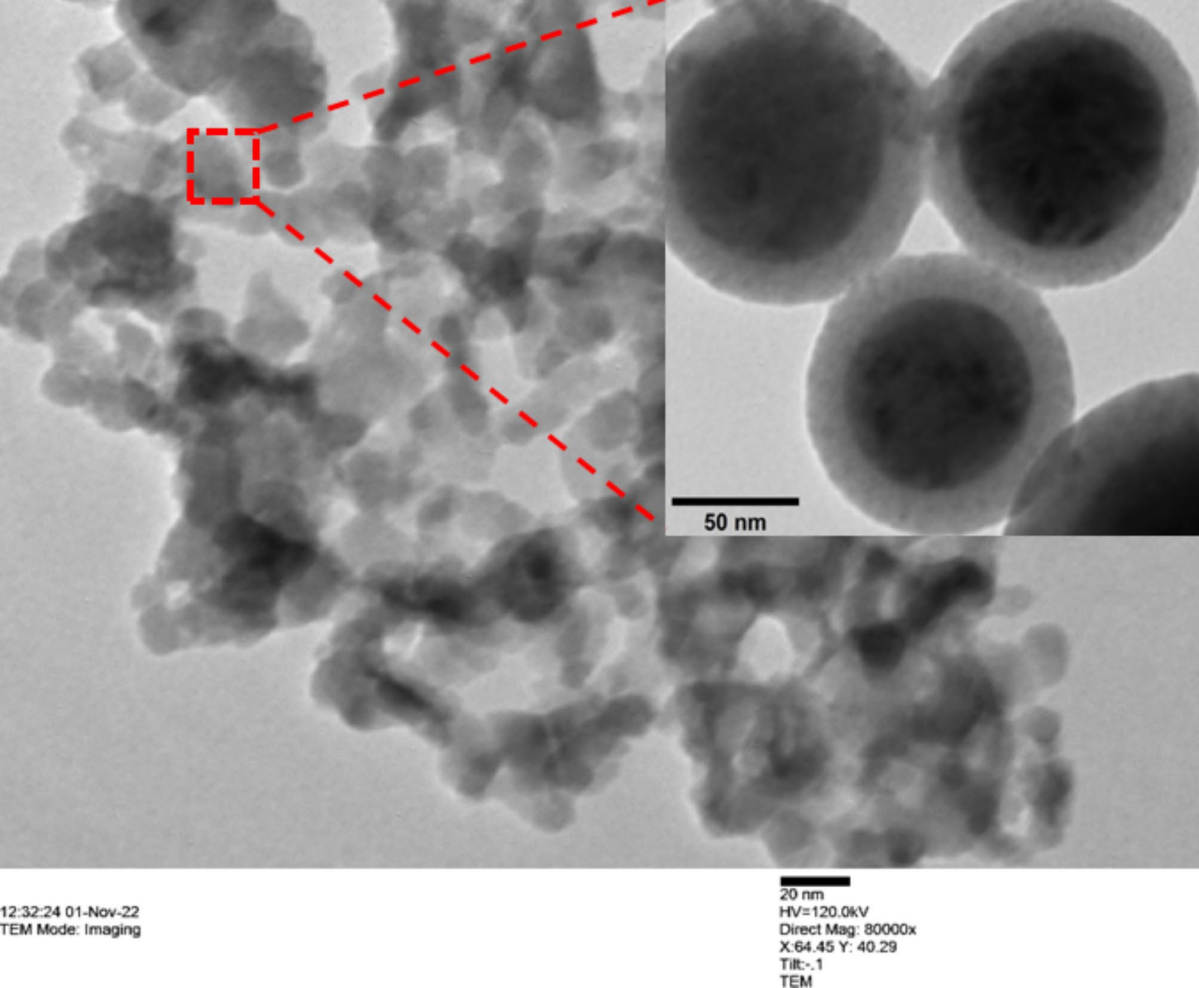
TEM image of alumina nanoparticles.

**Fig. 3:**
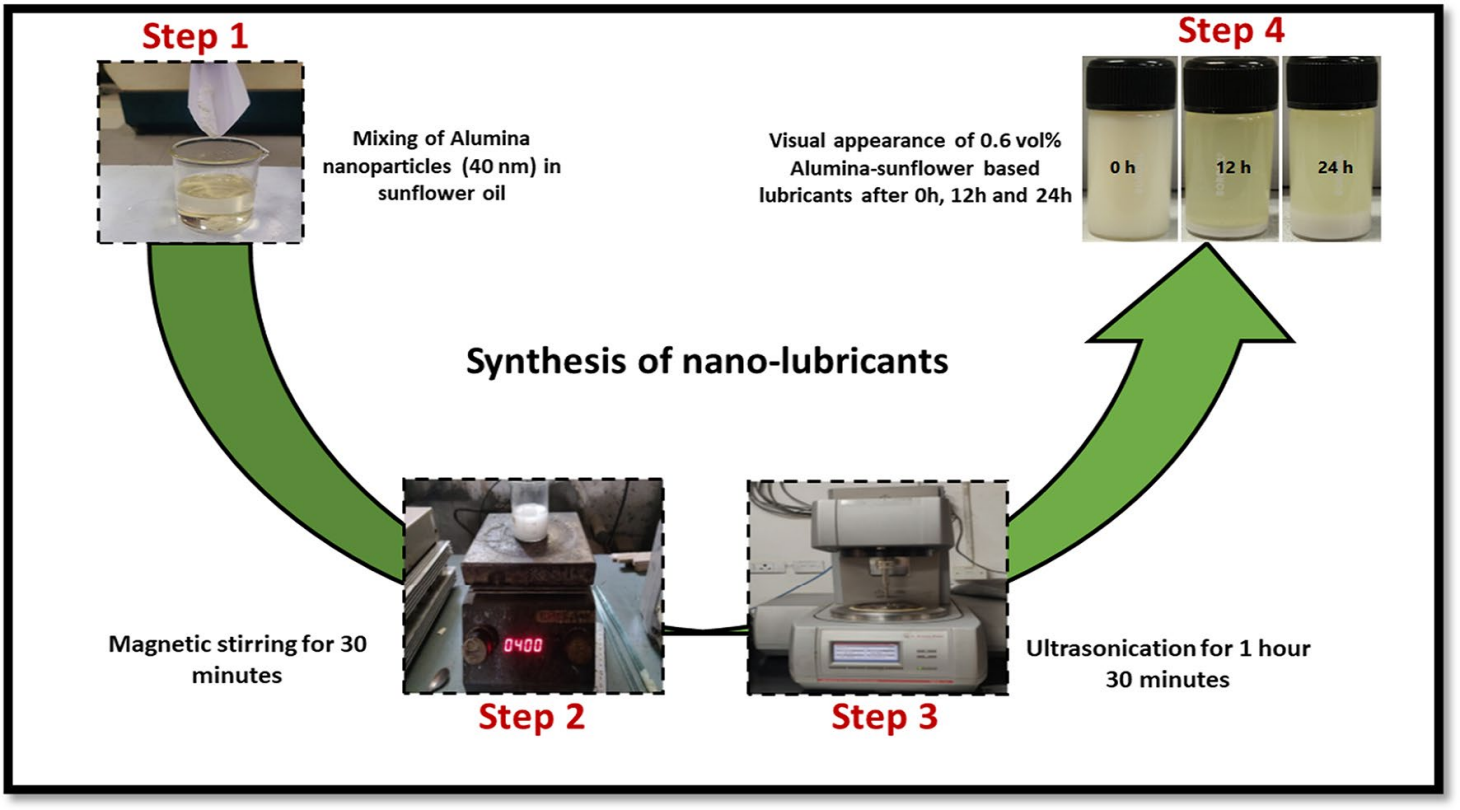
Steps involved in the synthesis of nano-lubricants.

For the current investigation, commercially manufactured alumina nanoparticles were sourced from Reinste Nano Ventures in New Delhi and utilized as an additive in sunflower bio-oil, which was purchased from Sri Poovathal Oil Mill in Tamil Nadu. The nanoparticles were approximately 40 nm in size and almost spherical in shape. The Transmission Electron Microscope (TEM) was used to capture the image of the Alumina nanoparticles, as depicted in Fig. 2. The development of Alumina-enriched sunflower bio-oil utilized nanoparticle concentrations of 0.2–1 vol%, with 0.2 vol% increments. A two-step method was adopted to make nanofluids samples, where purchased Alumina nanoparticles were mixed with the sunflower bio-oil by a magnetic stirrer at 700 RPM for 30 min. It took several rounds of stirring before a consistent dispersion of nanoparticles in the vegetable oil could be seen. Figure 3 elucidates a schematic representation describing the synthesis process employed for preparing Alumina-enriched sunflower bio-oil based lubricant. Nevertheless, a primary impediment preventing the commercialization of nanofluids resides in their inherent instability. The stability of these nanofluids is fundamentally governed by the intricate interplay and interactions among the nanoparticles present within the base fluids. The way nanofluids behaved depended on two forces that acted differently. One force pulled particles together, called the Van der Waals force, while the other force pushed them apart because of the electrical double layer^31–32^. The attraction force primarily encourages the accumulation of nanoparticles. As a result, a substantial fraction of the nanoparticles gathered and precipitated within the fundamental fluid medium. Furthermore, the formation of visible clusters and nanoparticle sedimentation is facilitated by gravitational force. In contrast, the aforesaid electrical force kept the nanoparticles apart. In the present study, the uniform dispersion of nanoparticles in sunflower bio-oil was achieved using a probe-type ultrasonicator (Model: Cromtech 200). The ultrasonicator operated at a frequency of 20 kHz, employing a pulse on-and-off cycle lasting 2 s, and the total duration of sonication was 2 h. To enhance the stability of nanoparticles, surfactants are commonly employed. In this study, surfactants were intentionally omitted during the preparation of the nanofluid due to sustainability concerns. Surfactants are often derived from petrochemicals, which can harm the environment and contribute to fossil fuel dependence. Their production is energy-intensive, leading to higher carbon emissions, and they can persist in ecosystems, causing long-term ecological damage. While surfactants help keep particles smaller than 10 nm stable and well-dispersed, they are not necessary for larger particles, like those used in this study. By omitting surfactants, the environmental impact is reduced while still maintaining the effectiveness of the nanofluid, supporting more sustainable practices.

### Characterization of prepared nano-lubricants

#### Wettability analysis

**Fig. 4:**
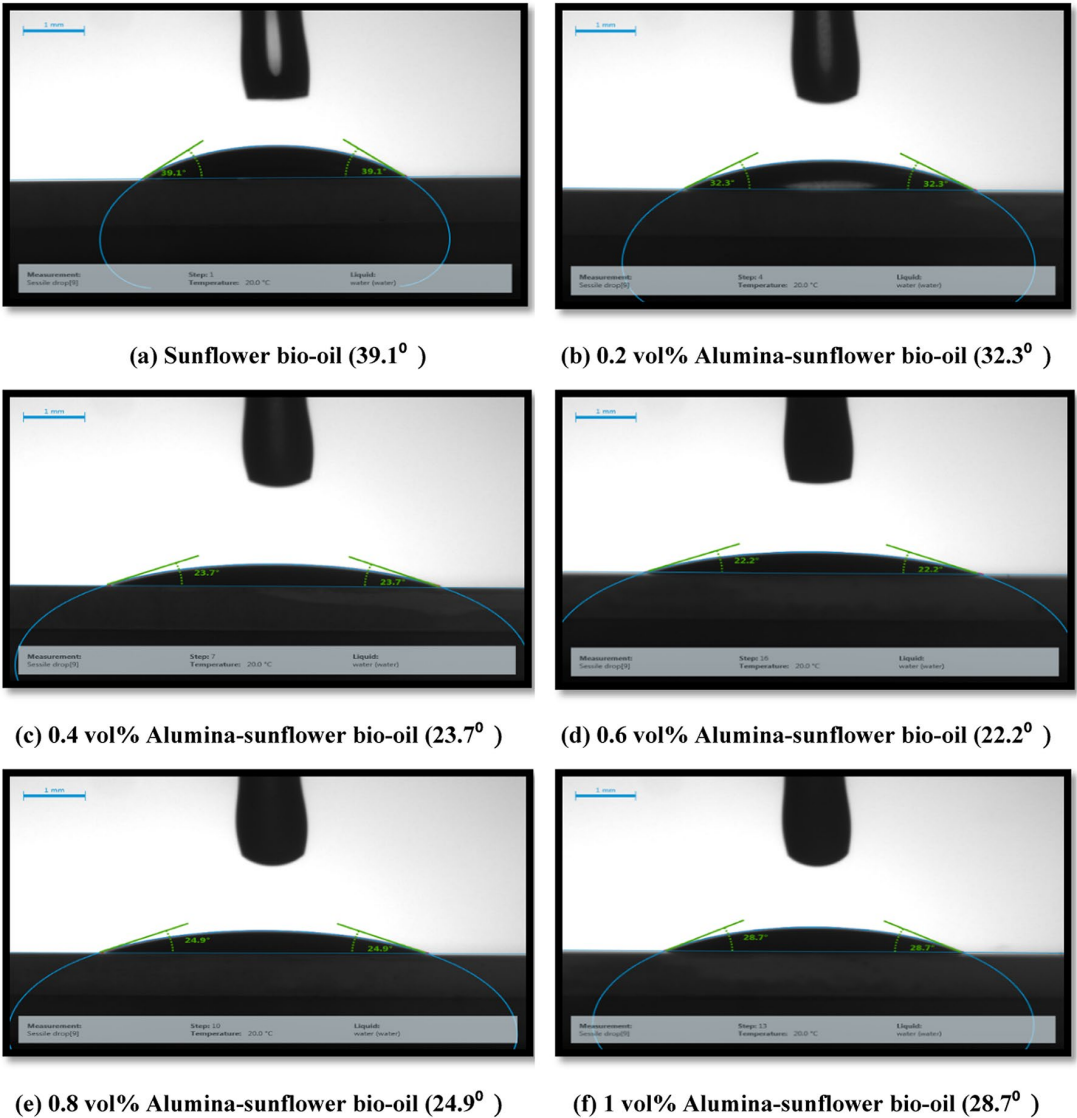
(**a**–**f**) Variation of contact angle with nanoparticle concentration.

Wettability pertains to the propensity of a lubricant to evenly disperse across the surface of a tool, thereby enhancing heat transfer across a wider area. The contact angle of the droplet is the result of the interplay between cohesive and adhesive forces at the interface. The equilibrium contact angle, signifying the state of minimal Helmholtz free energy, is achieved when the droplet attains its optimal spreading configuration. The free energy is determined by the potential interactions between fundamental atoms and molecules, primarily Vander Waal forces that affect surface tension. The aforementioned forces operate on a nanometric scale similar to the dimensions of nanoparticles^33^. Hence, the incorporation of nanoparticles into a liquid-solid-air interface is likely to exert an influence on the free energy. In the present investigation, a drop-shape analyzer (KRUSS DSA25) was employed to measure the contact angle exhibited by different nanofluid samples when in contact with glass surfaces, as illustrated in Fig. 4. In order to evaluate the wettability characteristics of the lubricants, the sessile-drop system was employed. Prior to conducting the test, the glass surfaces were thoroughly cleaned with acetone. Using a syringe equipped with a spring, droplets of lubricants (2 µL vol.) were carefully placed onto the glass substrate at ambient temperature. Figure 4 illustrates the enhanced wettability observed on the glass substrate as the nanoparticle concentration rises up to 0.6 vol%. The findings suggest that as the volumetric concentrations of nanoparticles increase, the contact angle decreases. This phenomenon can be ascribed to the influence of structural disjoining pressure, which facilitates the dispersion and spreading of nanofluids. However, beyond an Alumina concentration of 0.6 vol%, the contact angle begins to increase. It is understood that as viscosity takes precedence over disjoining pressure, the contact angle is inclined to rise with increasing nanoparticle concentrations.

#### Dynamic viscosity analysis

**Fig. 5:**
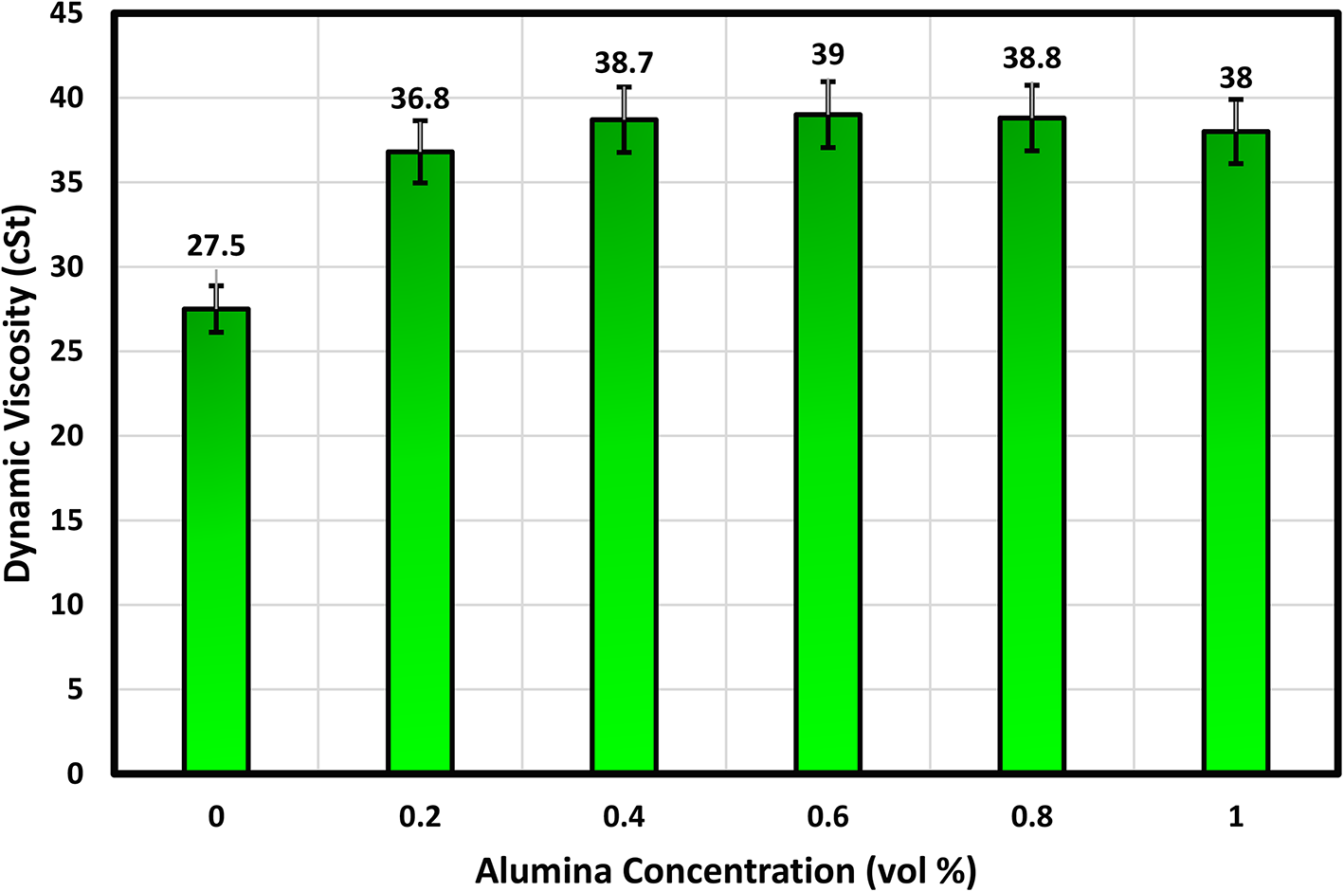
Variation of dynamic viscosity with Alumina concentration at 30 °C.

The lubricating efficacy of nanofluids is intrinsically linked to their viscosity characteristics. As such, an evaluation of the dynamic viscosity of in-house developed nano-lubricants was undertaken employing the Anton Paar^®^ rheometer (Model: MCR-102). A standardized sample volume of 7 ml was meticulously subjected to assessment for each individual nano-lubricant. The experimental procedures were executed at a room temperature of 30 °C while maintaining a consistent shear rate of 100 s^−1^. Figure 5 depicts the correlation between nanoparticle concentration and dynamic viscosity. As the nanoparticle concentration upsurges, the dynamic viscosity of the Alumina-sunflower bio-oil mixture follows an ascending trend until it reaches a peak at a concentration of 0.6 vol%. This viscosity increment is a consequence of the presence of nanoparticles, which introduce extra resistance to the flow, consequently raising the necessary torque to rotate the spindle^34^. However, beyond the 0.6 vol% concentration, a slight decline in viscosity value is observed. This phenomenon occurs due to excessive nanoparticle concentration in sunflower bio-oil, resulting in inadequate dispersion and sedimentation of nanoparticles^13^. Thus, dynamic viscosity diminishes after reaching a saturation point. Comparing it to pure sunflower bio-oil, sunflower bio-oil with 0.6 vol% Alumina exhibits a 41.82% increment in dynamic viscosity value.

### Machining details

**Fig. 6:**
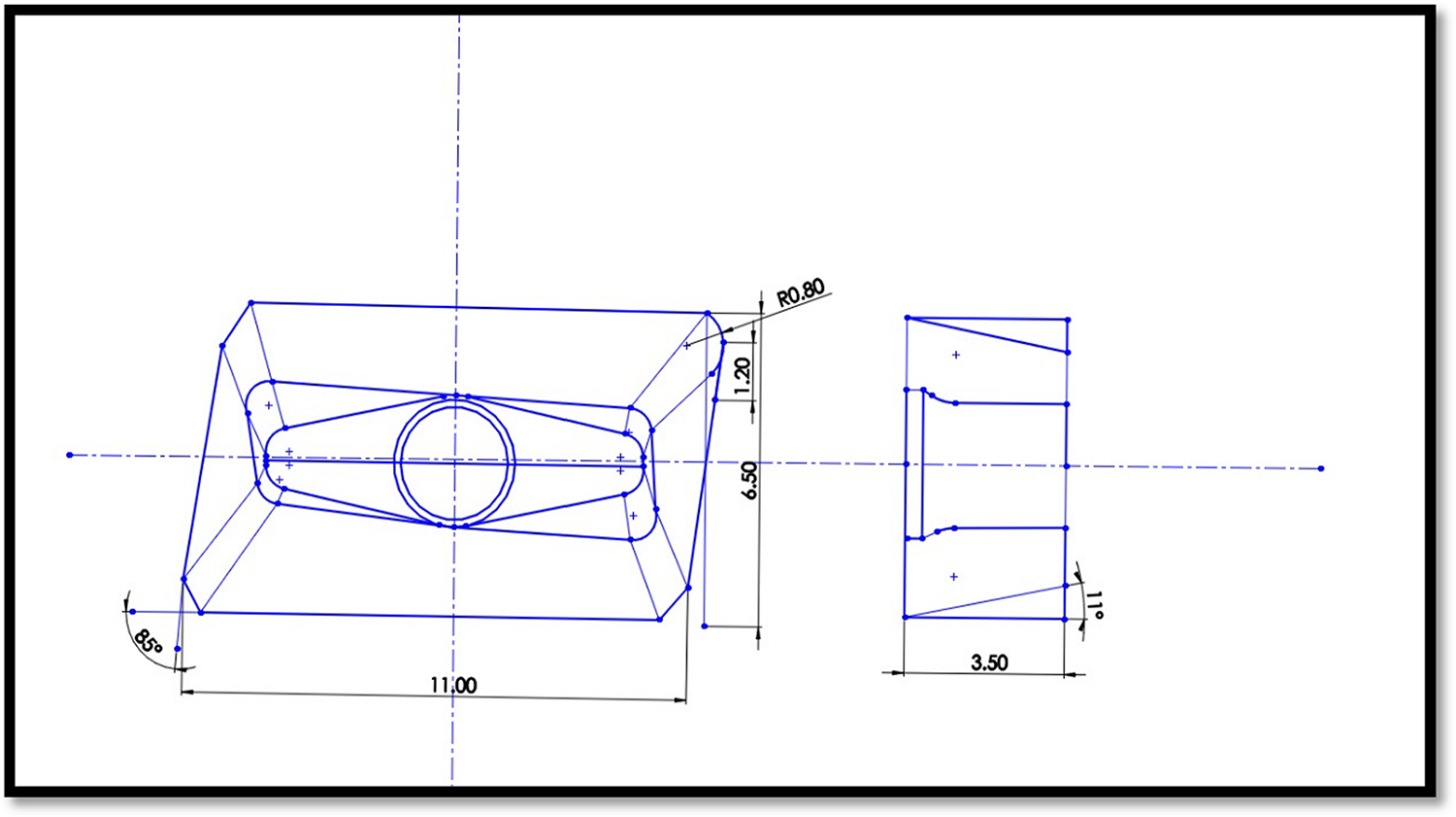
Geometry of the milling insert.

**Fig. 7:**
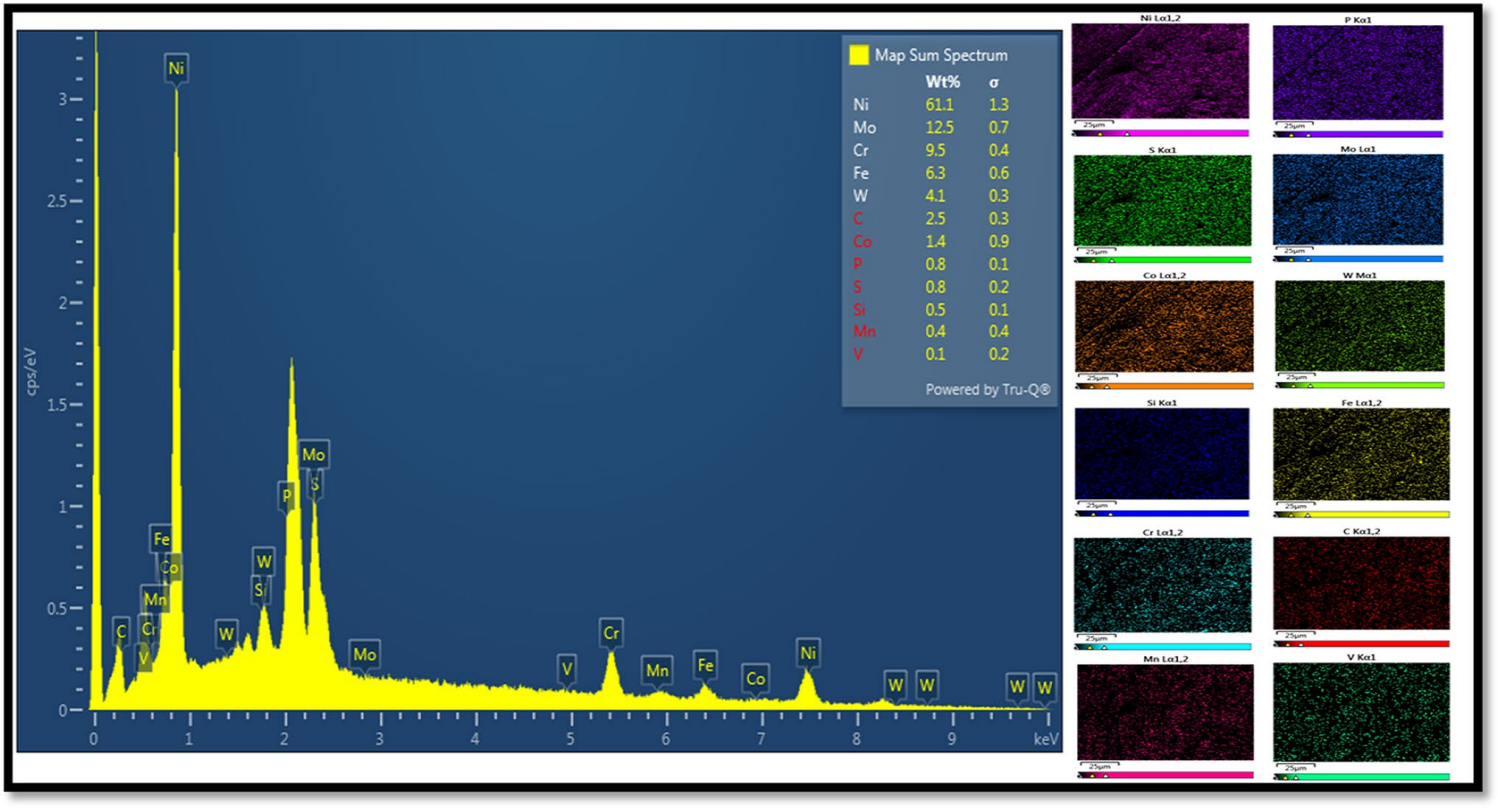
EDX analysis of Hastelloy C276.

The milling operations were executed in a CNC-assisted 3-axis milling machine (HARDINGE VMC 600 II). For each milling operation, a PVD AlTiN-coated carbide insert (MITSUBISHI, VP15TF) was utilized. The detailed geometry of the milling insert can be found in Fig. 6. The workpiece material used was commercially available Hastelloy C-276 alloy, with dimensions of 60 × 50 × 10 mm. Table 1 displays the various mechanical and thermal properties of Hastelloy C-276. Additionally, Fig. 7 illustrates the verification of the elements within Hastelloy C276 through EDX analysis. The milling operations were conducted in two distinct stages. The initial stage focused on assessing the efficiency of various lubricating mediums. The machining was executed in dry, compressed air, sunflower bio-oil, and a 0.6 vol% Alumina-sunflower bio-oil-assisted medium. A self-designed single-nozzle MQL system was used during experiments. The key parameters of the MQL system were kept constant, as specified in Table 2. Note that the MQL parameters for this study were chosen through an iterative process, using trial-and-error experiments within the manufacturer’s recommended range.

**Table 1:** The properties of Hastelloy C-276.

Property	Value
Density	8.89 g/cm^3^
Hardness	24 HRC (at 20 °C)
Melting range	1325–1370 °C
Thermal conductivity	10.1–12.5Wm^−1^ K^−1^ (at 23 °C)
Modulus of elasticity	205 GPa
Heat capacity	427 J/kg °C (at 20 °C)

**Table 2:** MQL set-up parameters.

Parameter	Value
Air pressure	6 kg/cm^2^
Flow rate	150 mL/h
Nozzle diameter	2 mm
Nozzle distance	30 mm
Nozzle angle	30°

The cutting parameters used for this stage were a cutting speed of 75 m/min, feed of 0.070 mm/tooth, and depth of cut of 0.3 mm. Following this, a set of sixteen experiments using the Taguchi L_16_ orthogonal array was conducted, with four levels and four factors, in order to identify the most environmentally sustainable machining conditions, taking into consideration technical, economic, and environmental parameters. The technical parameter assessed was surface roughness, and tool wear while the economic parameters included total machining cost. The environmental parameters considered were energy consumption and carbon emission. The design variables encompassed machining parameters such as speed, feed, depth of cut, as well as the lubricating mediums (dry cutting, compressed air, sunflower bio-oil, and 0.6 vol% Alumina-sunflower bio-oil). Note that the physical properties of alumina-enriched sunflower bio-oil demonstrated that the optimal volume percentage of Alumina nanoparticles in sunflower bio-oil is 0.6 vol% (in “Characterization of prepared nano-lubricants” section). This concentration threshold is crucial as it marks the point after which dynamic viscosity decreases and contact angle starts to increase. It is well-established that a higher viscosity value of nanofluids is essential for generating an improved lubricating film. Furthermore, a lower contact angle corresponds to enhanced wettability^1–35^. Consequently, 0.6 vol% alumina-sunflower bio-oil has been chosen for further comparative analysis. The experimental design is presented in Table 3.

**Table 3:** Experimental design.

Factor	Level 1	Level 2	Level 3	Level 4
Cutting speed (m/min)	45 (S_1_)	55 (S_2_)	65 (S_3_)	75 (S_4_)
Feed (mm/tooth)	0.01 (F_1_)	0.03 (F_2_)	0.05 (F_3_)	0.07 (F_4_)
Depth of cut (mm)	0.075 (D_1_)	0.15 (D_2_)	0.225 (D_3_)	0.3 (D_4_)
Lubricating medium	Dry (L_1_)	Compressed air (L_2_)	Sunflower bio-oil (L_3_)	0.6 vol% Alumina-sunflower bio-oil (L_4_)

**Fig. 8:**
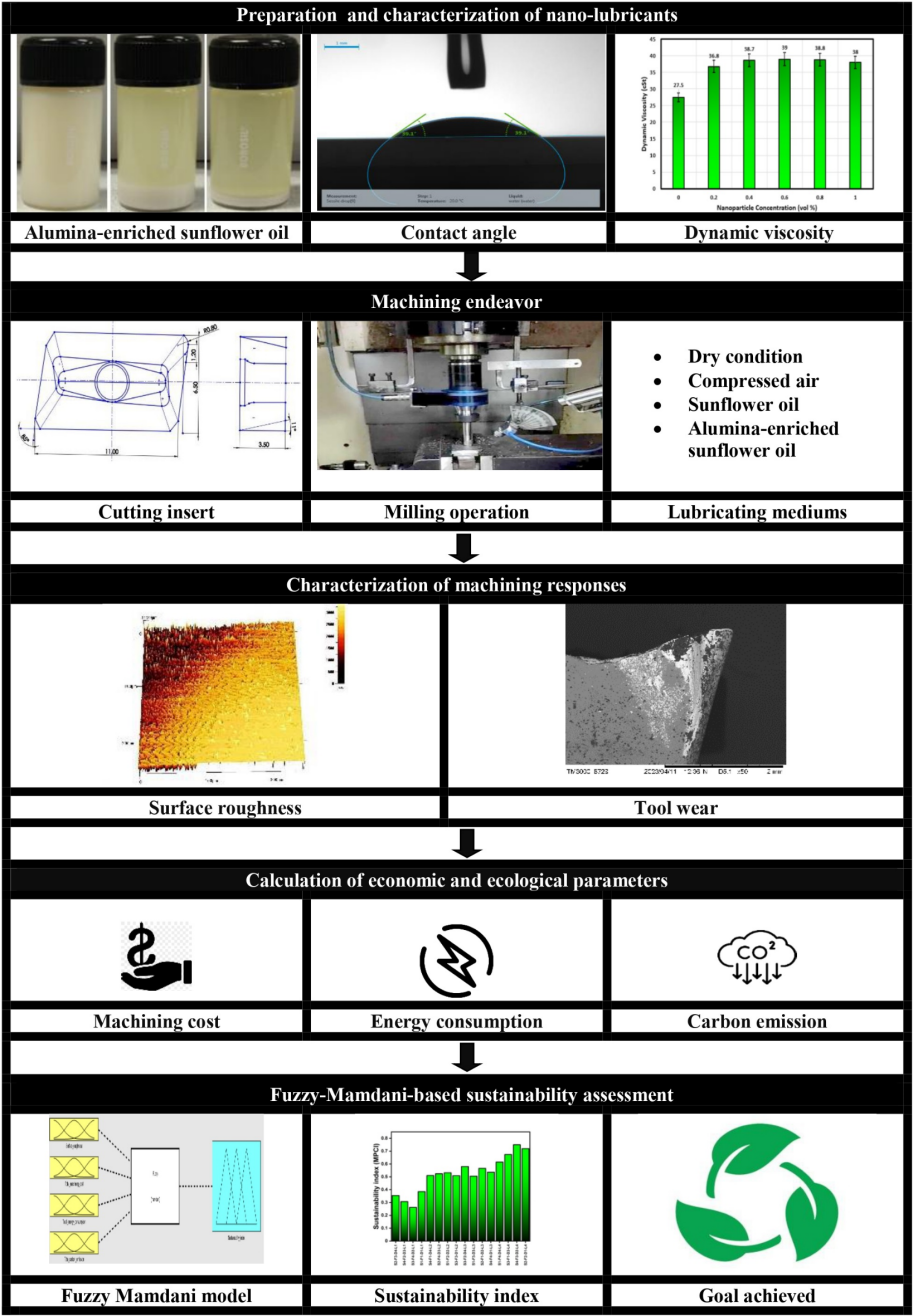
The flow diagram of the current study.

In this study, the determination of the average surface roughness (denoted as *R*_*a*_) was carried out employing a state-of-the-art 3D optical profiler, namely the KLA Tencor MicroXAM-100. The primary objective of this assessment was to gauge the precision of the machining operation. In order to maintain a high level of fidelity in the obtained measurements, the surface roughness was meticulously quantified at five distinct spatial locations, and the arithmetic mean of these measurements was subsequently computed. Subsequently, a comprehensive examination of the tool’s flank was conducted, employing an optical microscope (Model: ZEISS SteREO Discovery.V20). Due to the non-uniform wear patterns observed in the flank faces, the parameter of interest for evaluation was the maximum flank wear (*VB*_*max*_) rather than the average flank wear. Moreover, for an in-depth analysis of tool wear modes, an advanced scanning electron microscope, the HITACHI TM3000, was deployed. The key steps and procedures followed in this study are summarized in Fig. 8.

## Results and discussion

### UV-Vis spectrophotometry analysis

**Fig. 9:**
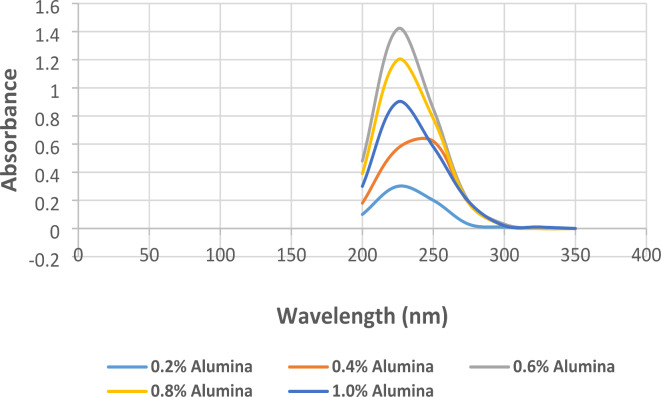
Variation of nanofluids absorbance with wavelength.

The physical stability of the sunflower bio-oil-based alumina nanofluid was evaluated using a UV–Vis–NIR spectrophotometer (Shimadzu UV-3600i Plus) to ensure accuracy in absorbance measurements, which could otherwise be affected by the high opacity of concentrated nanofluids. To avoid any potential discrepancies from opacity, the nanofluid samples were diluted at a ratio of 1 ml of nanofluid to 10 ml of base fluid. This approach provided a consistent basis for reliable spectroscopic analysis across different concentrations. The UV–Vis–NIR absorbance spectra for nanofluids at varying volume concentrations (0.2, 0.4, 0.6, 0.8, and 1.0 vol %) are presented in Fig. 9. A clear trend emerges, with the nanofluid at 0.6 vol % exhibiting a higher absorbance than those at 0.8 and 1.0 vol %, suggesting enhanced optical stability. This observation indicates a decrease in stability for the more concentrated samples, potentially due to increased sedimentation rates at 0.8 and 1.0 vol %, which can affect the dispersion of nanoparticles. This trend aligns with findings from Maheshwary et al.^36^, who reported a similar behavior in TiO₂-water-based nanofluids. According to their study, an increase in concentration leads to nanoparticle aggregation, resulting in larger effective particle sizes and diminished stability. Likewise, in the present study, it appears that a 0.6 vol % concentration optimally balances the stability and dispersion of alumina nanoparticles within the sunflower bio-oil-based nanofluid. Therefore, based on the stability and dispersibility observed, a 0.6 vol % concentration is deemed most suitable for further experimental work.

### Effect of lubricating mediums on machining response*s*

#### Effect on surface roughness

**Fig. 10:**
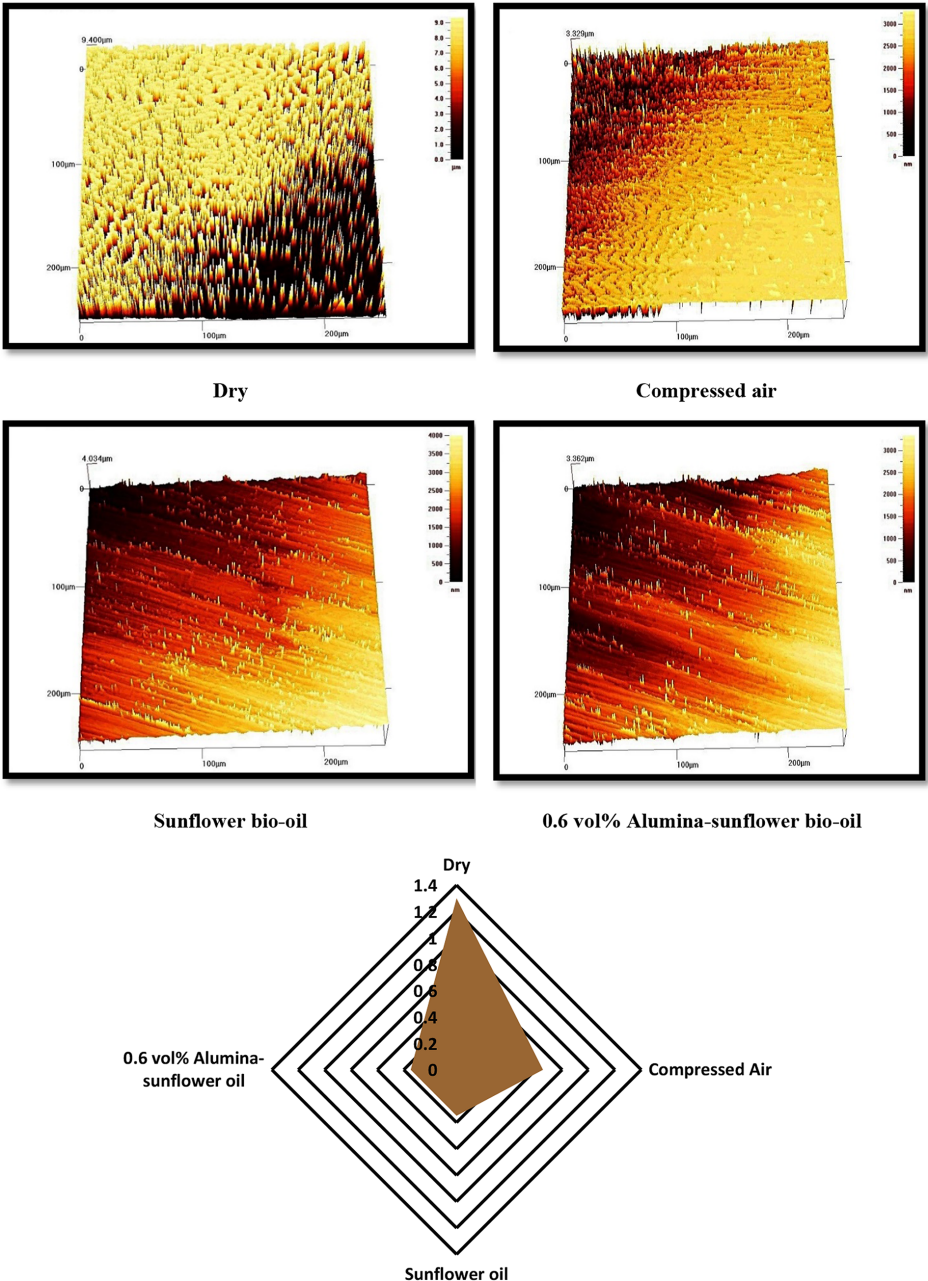
Variation of surface roughness in different lubricating mediums (S_4_-F_4_-D_4_).

The roughness of machined surfaces has a substantial influence on the performance, interactions between tools and workpieces, and the overall quality of the manufactured product. It is considered one of the most important measures in the production industry. Various factors, such as tool geometry, coatings, and lubrication environment, can affect surface roughness values. Here, an investigation was undertaken to elucidate the effect of various lubricating mediums on surface roughness. It is important to note that all pertinent machining parameters were diligently held constant throughout the experimental procedures. As stated earlier, a 3D profilometer was utilized to quantify the surface roughness values of the machined surfaces in different lubrication environments (Fig. 10). The outcomes of the investigation unveiled that the Alumina-enriched lubricant exhibited a noteworthy capacity to effectively penetrate into the cutting zone, leading to a discernible reduction in the surface roughness. This was further supported by the declining trend in cutting force and temperature values observed by the previous researchers^36–37^. As mentioned earlier, Alumina-enriched sunflower bio-oil exhibited higher viscosity compared to pure sunflower bio-oil, allowing it to persist in the machining zone for a longer time. Consequently, this lubricating medium reduced the *R*_*a*_ by 73.31%, 47.02%, and 0.57% compared to dry, compressed air, and sunflower bio-oil-based mediums, respectively. The minimal difference in surface roughness between sunflower bio-oil-based lubricants and Alumina-enriched sunflower bio-oil-based lubricants in machining operations can be attributed to the effective lubricating properties of both. Sunflower bio-oil-based lubricants possess decent lubricity, reducing friction and heat during machining. The addition of Alumina nanoparticles further enhances lubrication and provides a protective boundary between the cutting tool and the workpiece. This results in reduced tool wear and improved surface finish. Consequently, the marginal variance in surface roughness reflects the similar lubricating capabilities of both mediums, emphasizing the effectiveness of sunflower bio-oil-based lubricants even without nanoparticle reinforcement. In this context, the research findings of Gajrani et al.^13^ hold significance. The authors demonstrated the formation of a tribo-film on machined surfaces using both vegetable oil and nanofluid mediums. This ultimately led to the creation of smoother machining surfaces.

#### Effect on tool wear

**Fig. 11:**
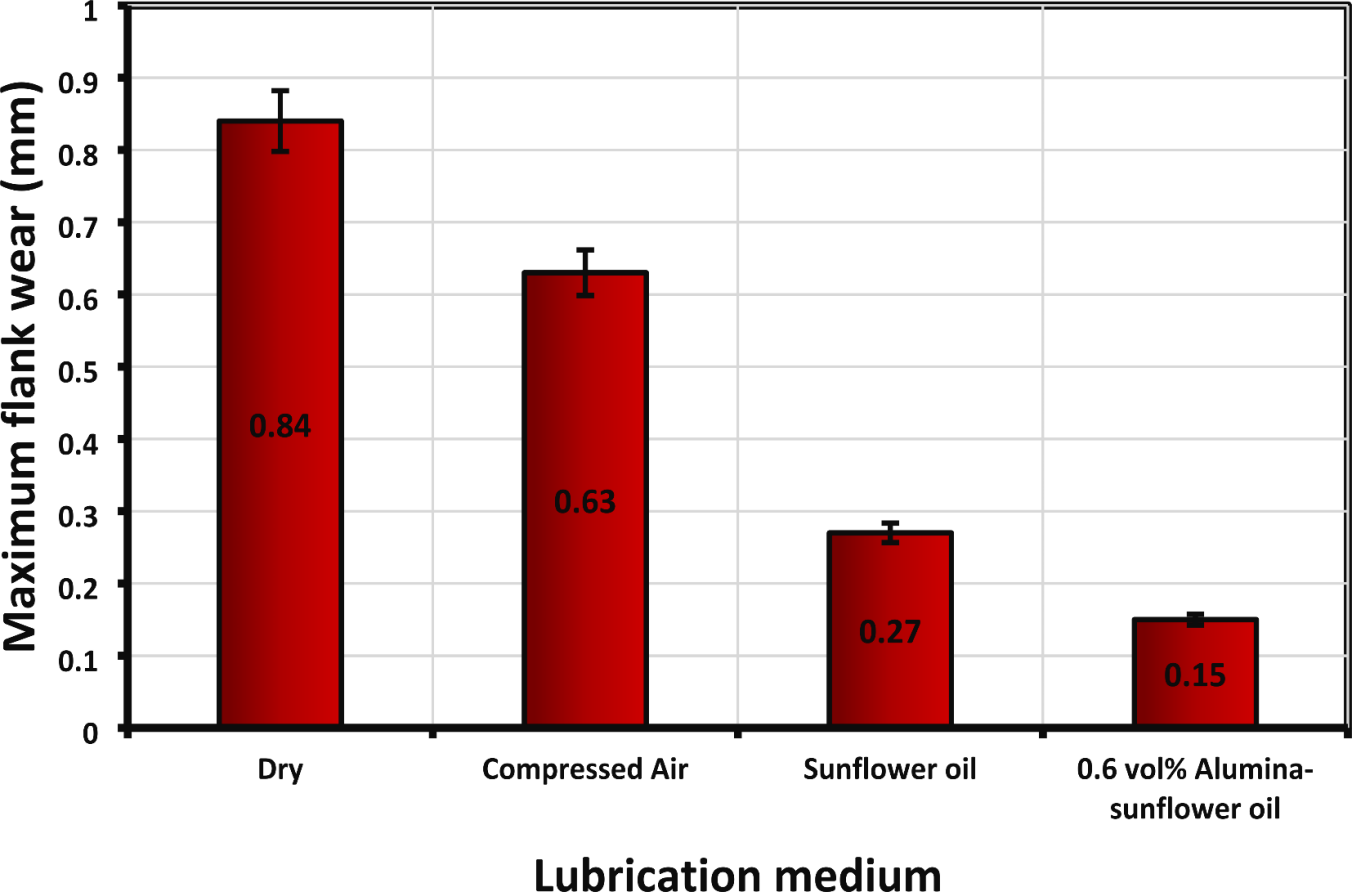
Variation of maximum tool wear values with lubricating medium (S_4_-F_4_-D_4_).

Tool wear is a vigorous factor in machining that has a noteworthy impact on machining quality and cost^38^. The objective of the study is to scrutinize the wear behavior of AlTiN-coated inserts and assess the lubricating performance of various mediums, including dry cutting, compressed air, sunflower bio-oil, and 0.6 vol% Alumina-sunflower bio-oil. Here, the input parameters were kept constant while the lubrication mediums were varied. Every cutting operation lasted for 25 min, and the *VB*_*max*_ values were measured using a microscope. The results revealed that the minimum quantity of Alumina-sunflower bio-oil effectively reduced tool wear by minimizing friction between the tool and the workpiece. Figure 11 visually illustrates the variation of *VB*_*max*_ values with different lubrication mediums, highlighting the superior lubricating capability of the Alumina-sunflower bio-oil-based medium compared to the others. Specifically, the 0.6 vol% Alumina-sunflower bio-oil medium decreased wear values by 82.14%, 76.19%, and 44.44% compared to dry cutting, compressed air, and sunflower bio-oil mediums, correspondingly. However, it is important to acknowledge previous research, such as the study executed by Şirin et al.^16^, which emphasized the influence of nanoparticle concentration on tool wear. The authors observed that increasing the concentration of nanoparticles positively affects tool life up to a certain threshold. However, beyond this saturation limit, an adverse effect occurs as the nanoparticle concentration exceeds a specific limit. The excessive accumulation of nanoparticles results in increased viscosity of the base fluid, thereby reducing its flowability. This change in the characteristics of the nanofluid leads to stickiness and a decline in lubricating properties. Consequently, the friction between the tool and the workpiece intensifies, resulting in a reduction in the tool’s longevity. Moreover, higher levels of nanoparticle reinforcement raise the probability of nanoparticle agglomeration. Hence, it is crucial to determine the optimal volumetric concentration of nanoparticles to attain superior lubricating properties.

**Fig. 12:**
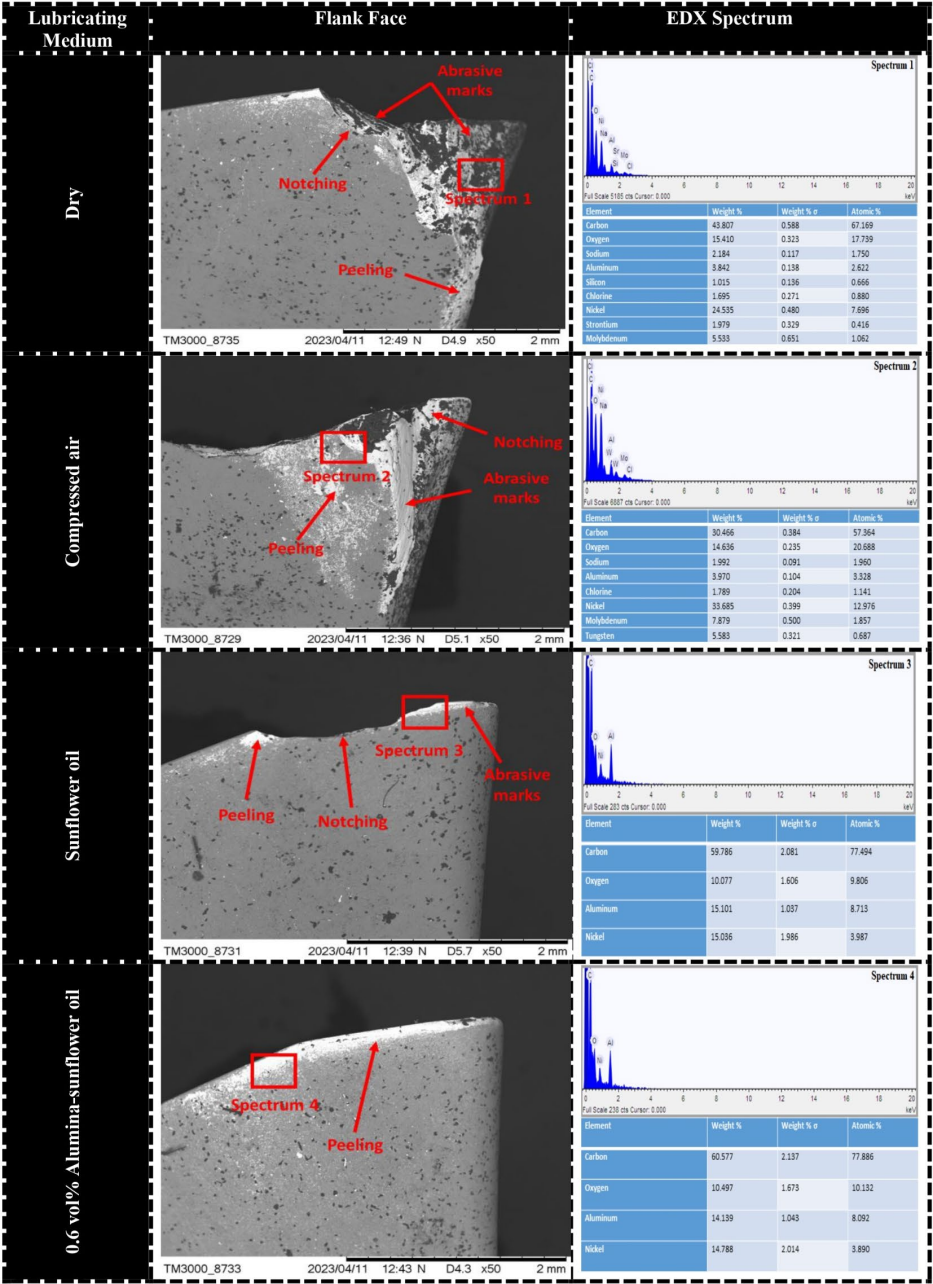
SEM images of tool flank in different lubricating mediums (S_4_-F_4_-D_4_).

Flank wear plays a crucial role in assessing materials’ machinability since it directly affects the quality of the machined surfaces. However, accurately measuring flank wear becomes more challenging due to factors like adhesion and thermal cracking, which result in non-uniform wear patterns. In the case of Hastelloy C-276 machining, notch wear has been recognized as the primary reason for flank failure, as seen in Fig. 12. Notch wear extends further towards the principal cutting edge area under severe conditions such as dry and compressed air, while sunflower bio-oil and Alumina-enriched sunflower bio-oil-based mediums help limit notch wear nearer to the tool nose area. Notch wear is commonly observed in superalloy machining and is influenced by interdependent factors. Previous studies by Li et al.^39^ highlighted that significant adhesion of workpiece material is responsible for severe notch wear. To investigate this further, an energy-dispersive X-ray spectroscopy (EDX) analysis is performed, which confirms the adhesion of workpiece material to the cutting tool through the presence of elements like nickel on the fractured cutting edge. On the contrary, the high-work hardening behavior of nickel-based alloys has been identified by various researchers as a contributing factor to notch wear^40–41^. The pronounced work hardening characteristic leads to unstable cutting forces during machining, thus, increasing the likelihood of tool fracture in the case of superalloys.

Under dry-cutting conditions, the tool flank face experienced micro-chipping due to the rapid movement of strain-hardened chips, resulting in high abrasion. Previous research conducted by Sen et al.^42^ has confirmed that carbide particles in nickel alloys can cause cracks, scratches, and grooves on the tool. Conversely, the utilization of nano-MQL during machining produced superior results compared to other lubricating methods. Examination of the SEM micrograph revealed minimal peeling of coated material on the principal cutting edge, indicating enhanced tool performance. This enhancement can be rationalized through the contact angle theory, wherein the reduced contact angle exhibited by Alumina-enriched sunflower bio-oil droplets facilitates superior surface wetting, thereby prompting the formation of a lubrication film.

While nano-MQL has shown significant improvement in tool performance, compressed air, and sunflower bio-oil have also proven partially effective in reducing abrasive wear. Compressed air serves as a cooling medium, diminishing heat generation at the cutting zone and thereby minimizing thermal-induced wear. Additionally, its high-pressure application removes chips efficiently, reducing friction and the likelihood of abrasive damage on the tool’s surface. Sunflower bio-oil, aids in reducing abrasion due to its superior lubricating properties. By enhancing the wetting of the tool-workpiece interface, sunflower bio-oil promotes a protective film formation, effectively lowering friction and wear. This lubricating layer acts as a barrier against direct contact between the tool and the work material, leading to reduced tool wear. Notably, recent investigations by Kong et al.^43^ indirectly corroborate the aforementioned theoretical proposition. Their research underscores the efficacy of surface wetting via rapeseed oil application, achieved through brush-based methods, resulting in diminished notch wear compared to both dry and wet machining processes.

**Fig. 13:**
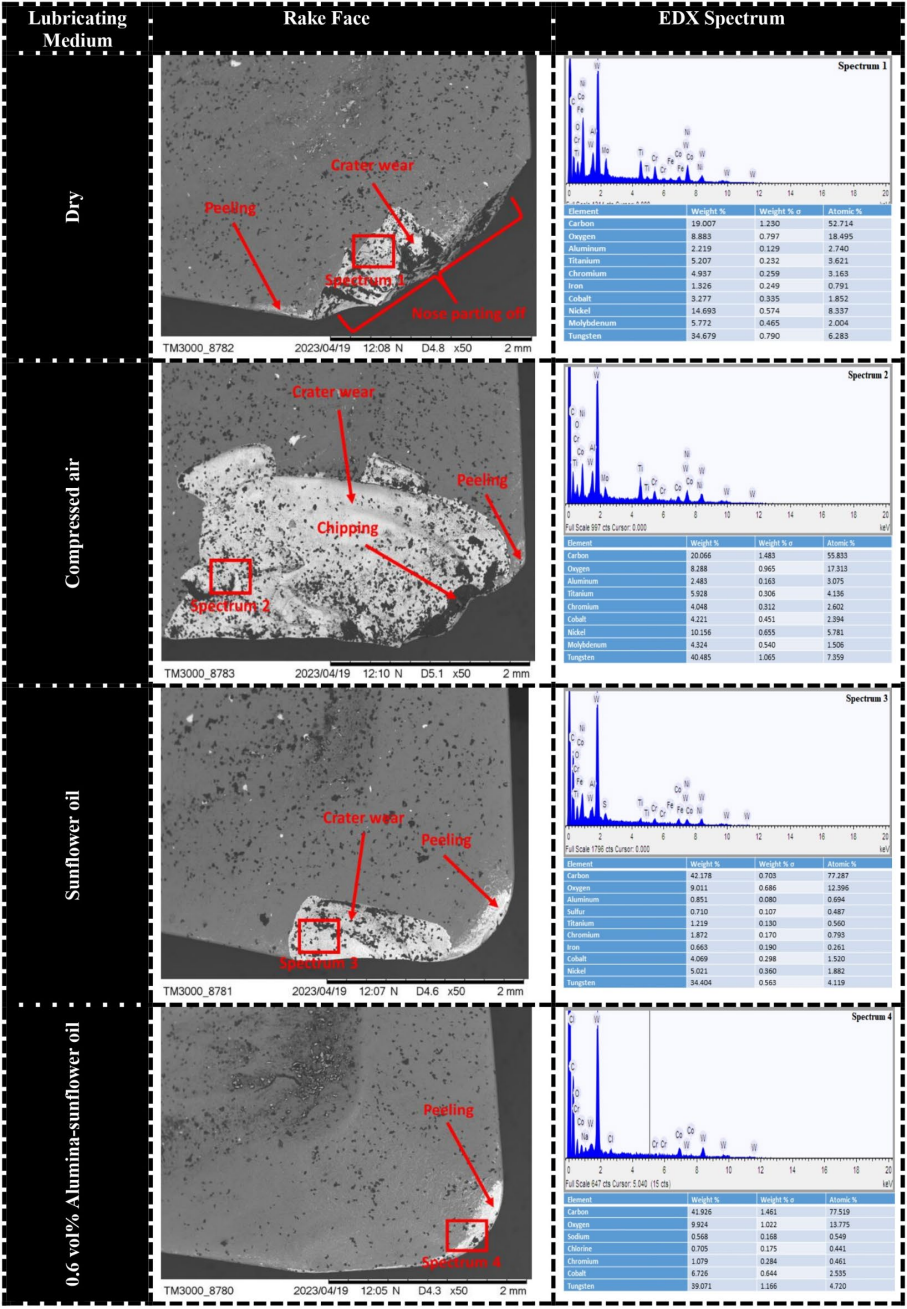
SEM images of tool rake in different lubricating mediums (S_4_-F_4_-D_4_).

In the course of machining operations, the interplay between chips and the tool can give rise to substantial damage on the rake surface, primarily attributable to escalated contact friction and elevated cutting temperatures. These factors result in higher cutting forces and increased power consumption. Consequently, it is necessary to comprehend the wear mechanism occurring on the rake surface in the machining of Hastelloy C-276. SEM micrographs depicted in Fig. 13 were captured using different lubrication mediums to investigate the wear mechanisms. In dry conditions, the predominant wear detected on the rake face was a significant tip fracture. Conversely, when utilizing compressed air, the sticking of chip fragments was observed. Both dry and compressed air conditions demonstrated coating peeling as the primary wear mechanism on the rake surface. However, when employing 0.6 vol% Alumina-sunflower bio-oil based lubrication, only mild peeling of the coating was observed. An EDX analysis provided confirmation of the adhesion of nickel-based superalloy to the tool rake. The occurrence of chipping, fracture, and peeling under dry and compressed air conditions can be attributed to attrition wear and thermal cracking, which are caused by high temperatures. The unique characteristics of nickel-based alloys, for instance, thermo-mechanical fatigue, irregular chip flow, and fluctuating cutting forces contribute to attrition wear. Additionally, the adhesion of work material plays an important role in chipping, fracture, and peeling by facilitating the accumulation of a built-up edge (BUE) on the rake face. Nevertheless, because of the dynamic characteristics inherent in the machining process, the BUE undergoes instability and is consistently eliminated from the rake face. Consequently, owing to the repetitive occurrence and elimination of the BUE, the rake surface undergoes chipping, fracturing, and the peeling of its protective coating^44^.

Moreover, notable crater wear was identified in both dry and compressed air-based mediums. Chetan et al.^45^ stated that the presence of craters on the tools rake can be elucidated by the diffusion mechanism. In challenging cutting conditions, elevated temperatures on the rake surface provide sufficient energy for carbon diffusion from the carbide. Nevertheless, the use of nanoparticle-enriched vegetable oil-based lubrication resulted in minimal crater wear on the rake surface of the cutting tool. The improved performance of the cutting insert in this scenario can be attributed to the remarkable surface tension exhibited by the specially formulated nano-lubricant. A lower surface tension value indicates enhanced spreadability of the fluid across the mating surfaces. Consequently, the utilization of MQL and Nano-MQL mediums demonstrates superior results in reducing rake face wear in the milling of Hastelloy C-276.

#### Development of the surface protection film

**Fig. 14:**
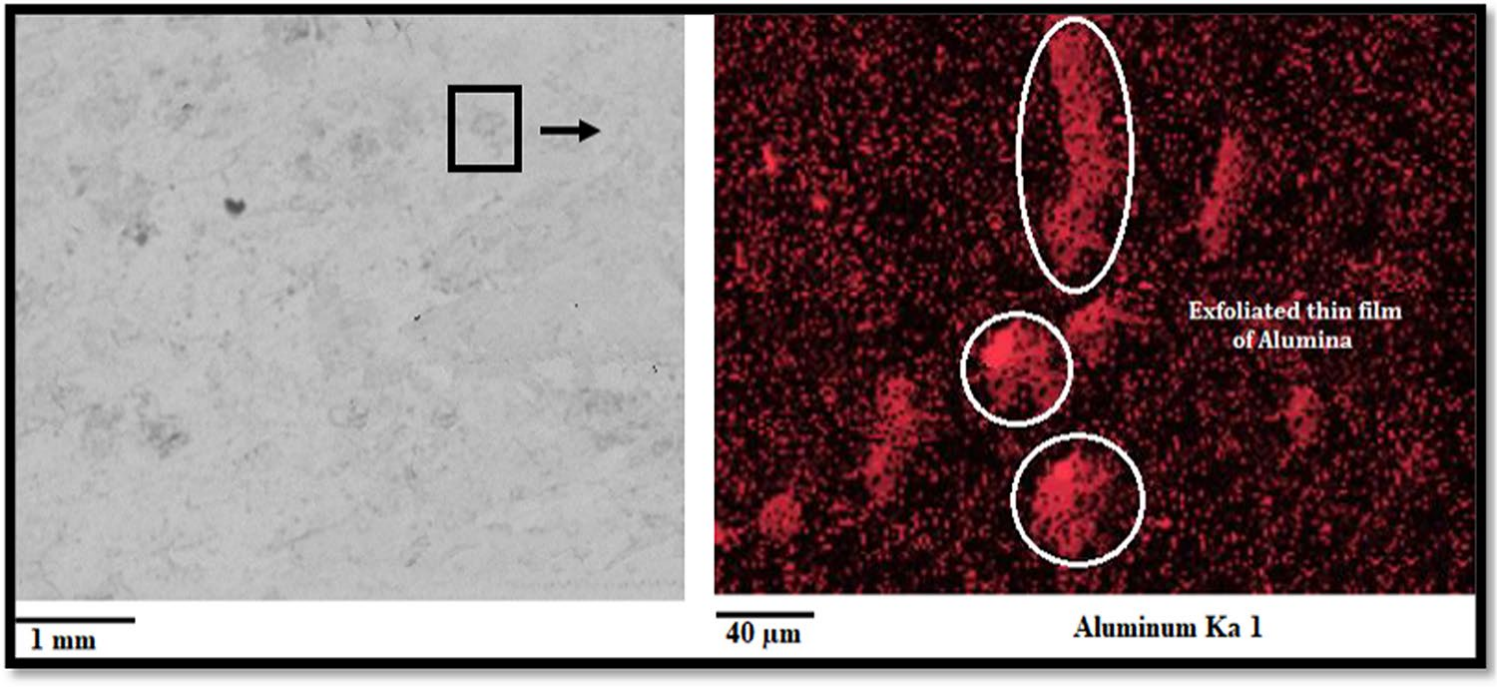
Distribution of Alumina nanoparticles in the machined surface.

The surface elemental mapping of the machined surfaces under the nano-lubricating environment is shown in Fig. 14. These images show that thin surface protecting films were developed on machined surfaces, and contain billions of Alumina. The formation of thin exfoliations or films may occur because the optimum concentration of nanoparticles increases sunflower bio-oil’s viscosity. Thus, most nanoparticles present in the tool-work interface may serve as a spacer and moderate the contact friction of the tool-workpiece. The porous nature of most nanoparticles imparts a notable elasticity, enhancing resilience within a specific loading range and modulating the clearance between tool-workpiece interfaces. The high pressure of the deposited nanoparticles and the gap between the tool-work interfaces may result in high contact resistance, which was responsible for forming the protecting layer on the machined zone through a chemical reaction. Besides, the elevated heat generated in the tool-work interface transformed the elasto-hydrodynamic lubrication into boundary lubrication which also facilitates the formation of a thin film. During machining operation, a significant number of alumina nanoparticles engage in frictional interactions with the asperities present on the work surface; thus, a fresh metallic surface was exposed to the nanofluid. Therefore, strong chemical reactions were taken between the newly developed surface and the nanoparticles present in the cutting oil. This phenomenon may be attributed as a contributing factor to the formation of the tribo-film. As a result, the morphology of the cutting surface was certainly increased, and the coefficient of friction was reduced^46^.

**Fig. 15:**
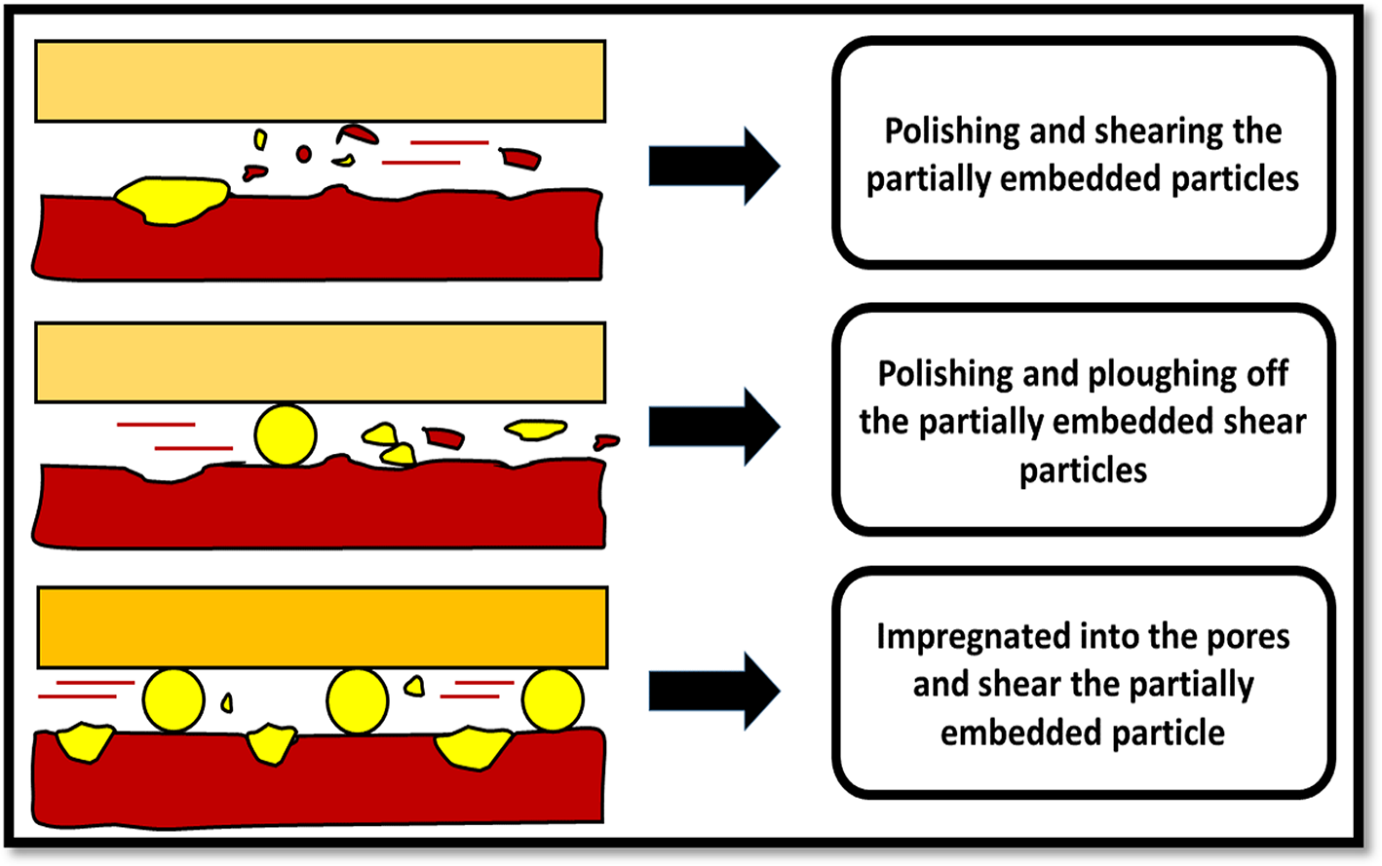
The mechanism of nanoparticles seems to assist the machining operation.

The cutting operation performed by the nano-sized particles can be classified into three levels (Fig. 15). Primarily, the nanoparticles present in the cutting oil were embedded into the machining zone. Because of the elevated pressure of the machining zone, most nanoparticles collide with the asperities. Following this, the nanoparticles underwent deformation and a modification in shape due to compression. The shared nanoparticles continued to contribute to the cutting process, albeit not as effectively as their unshared counterparts, which retained their spherical form and exhibited a low coefficient of friction while traversing the cutting surfaces. In some parts of the machined surface, billions of nanoparticles are concentrated, ploughed off by the fresh nanoparticles, and both continue the polishing process. Nanoparticles that are ploughed off during the process leave behind a thin film on the contact area due to the substantial damage from severe loading. Ultimately, these nanoparticles become impregnated into the pores of the machined surface, while incoming nanoparticles engage in shaving off the partially embedded ones. The rolling action of these nanoparticles generates surface asperities and facilitates the creation of an oil film that is readily sheared. In this manner, the machined surface undergoes a polishing effect, resulting in an enhanced and superior surface morphology^47^.

### **Effect of lubricating mediums on economic and ecological factors**

#### Effect on machining cost

Indeed, achieving an economically efficient machining process plays a pivotal role in promoting sustainable production. To achieve this goal, a comprehensive examination of the entire cost spectrum, spanning from the initiation to the culmination of the machining operation, is imperative. The total cost of machining denoted as *C*_*T*_, encompasses three key components: the complete expenditure on electricity consumption (*C*_*E*_), market price of cutting inserts (*C*_*I*_), and the comprehensive cost of lubricants (*C*_*L*_), as detailed in Eq. (1). Nevertheless, an attempt can be made to calculate the total machining cost, taking into account both overhead and disposal costs. In this study, the choice to disregard overhead and disposal costs could be connected to a simplified initial assessment strategy. For rapid estimates, concentrating solely on direct costs such as electricity, tool, and lubricant expenses offers a clear view of the immediate costs involved in the machining process. Moreover, overhead and disposal costs may not be seen as directly related to the particular machining process. Even though neglecting them may simplify the calculation, a comprehensive analysis should factor in all pertinent costs to ensure an accurate decision.

1 $$C_{T} = C_{E} + C_{I} + C_{L}$$

*C*_*E*_ can be described by the Eq. (2).

2 $$C_{E} = \frac{Unit \; Energy \; Cost}{{{\upeta } \times 60 \times 1000}} \times T_{M} \times \left( {Cutting \; power + Standby \; power} \right)$$

In India, the average unit cost per kilowatt-hour of energy is Rs. 6.19, where the machine efficiency (typically 85%) is denoted as η, and the cutting power of the machine tool is 2216 W (at a speed of 100 m/min), with the machine’s constant standby power consumption at approximately 1987 W, the machining time (*T*_*M*_) is determined using Eq. (3).

3 $$T_{M} = \frac{Length \; of \; cut + Machining \; allowance}{{Feed\; rate \times RPM}}$$

*C*_*I*_ can be calculated using Eq. (4):

4 $$C_{I} = \frac{Market\; price\; of \; cutting \; insert}{{Tool\; life}} \times T_{M}$$

In machining, when the cutting insert reaches a wear level of 0.3 mm, it is deemed unfit for further use. The initial cost of the insert was Rs 850.

The *C*_*L*_ can be expressed by Eq. (5):

5 $$C_{L} = Lubricant\; cost \times Flow\; rate\; of \; MQL \times T_{M}$$

In this context, the price of sunflower bio-oil per liter stands at Rs 250, while the cost of Alumina nanoparticles per gram is Rs 90, and a constant MQL flow rate of 150 mL/h is maintained during the entire experiment.

**Fig. 16:**
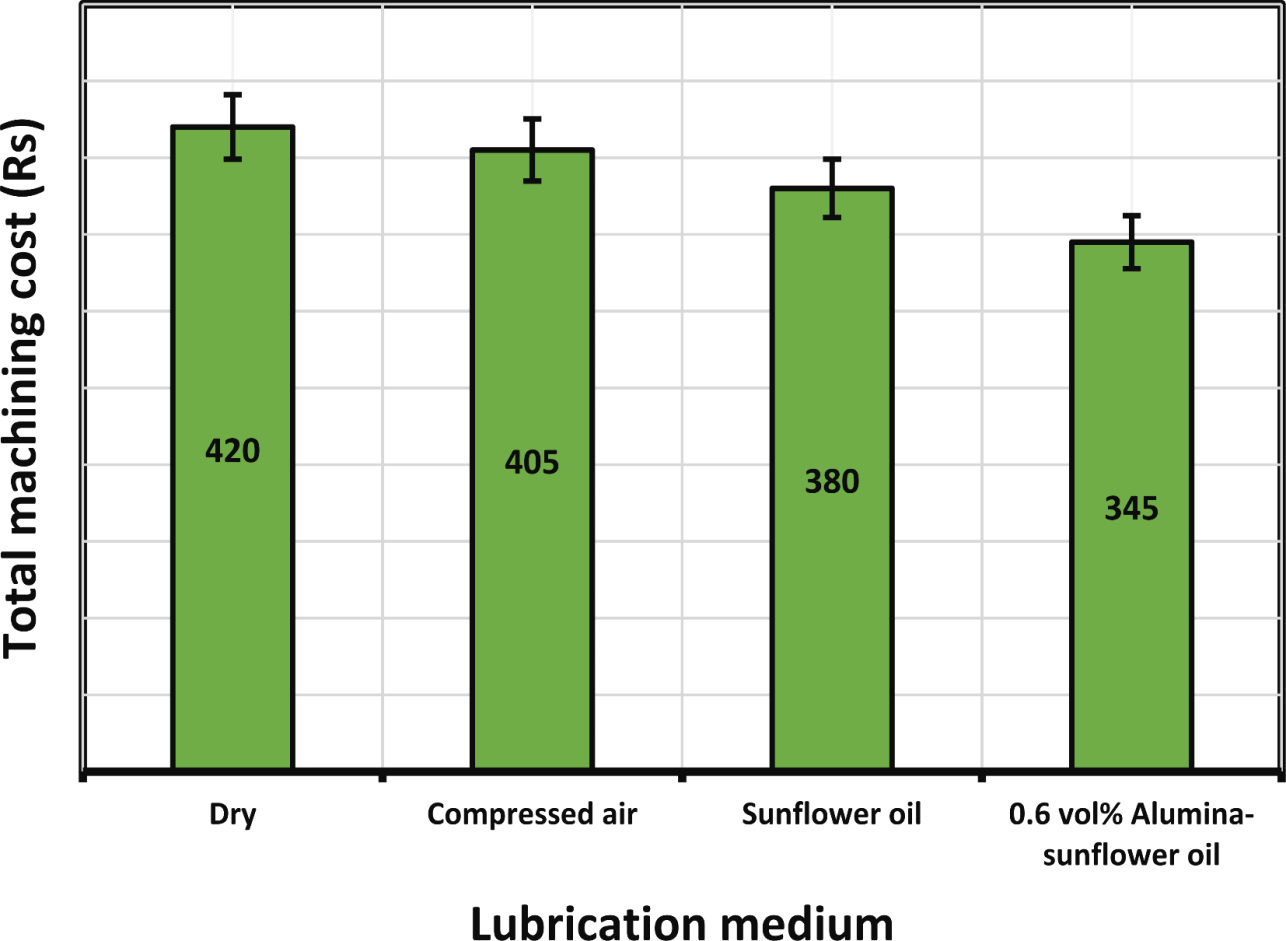
Total machining cost values in different lubrication medium.

Figure 16 presents an illustration of the overall machining costs within various lubricating environments, including dry conditions, compressed air, sunflower bio-oil, and 0.6 vol% Alumina-sunflower bio-oil. It is noteworthy that the use of a 0.6 vol% Alumina-sunflower bio-oil was found to require approximately 3.57%, 9.52%, and 17.86% less machining cost than dry, compressed air, and sunflower bio-oil-based machining. This observation aligns with prior research findings, which have consistently highlighted the advantages of utilizing nano-lubricants in milling processes. These advantages stem from its superior lubricating and heat-dissipating properties. The introduction of Alumina nanoparticles into the oil matrix significantly augments its lubricity, thereby reducing friction and wear on cutting tools. This results in a decreased need for frequent tool replacements, ultimately leading to reduced tool-related expenses. Furthermore, the remarkable heat transfer characteristics of Alumina-sunflower bio-oil enhance machining efficiency, which translates to a decrease in electricity consumption costs. However, it is important to note that when considering the lubricant cost, 0.6 vol% Alumina-sunflower bio-oil entails higher expenses due to the incorporation of Alumina particles. Alumina, as a high-performance ceramic material, incurs substantial manufacturing costs. Additionally, the production of the resultant mixture may necessitate rigorous quality control measures to ensure consistent and reliable performance. In contrast, the use of dry machining, compressed air, and sunflower bio-oil proves to be more straightforward and cost-effective for milling operations. In the study conducted by Gupta et al.^48^, it was observed that electricity consumption constitutes approximately 45–50% of the overall machining expenditure. Consequently, in order to mitigate electricity consumption, manufacturers have the option to make trade-offs with lubricant cost and opt for nanoparticle-enhanced lubricants when machining superalloys.

#### Effect on energy consumption and carbon emission

Several factors underscore the importance of energy consumption analysis: (a) Nearly half of production expenses stem from energy costs. (b) The ongoing global quest for alternative energy sources. (c) The detrimental environmental impact of carbon emissions resulting from energy consumption. This study, utilizing Eq. (6), investigated energy consumption to create a more energy-efficient cutting environment.

6 $$E_{C} = \frac{{F_{R} \times V_{C} \times T}}{1000}$$

where *F*_*R*_ is the resultant cutting force (N), *V*_*C*_ is cutting speed in m/min, *T* is cutting time in min and *E*_*C*_ is energy consumption in kJ.

**Fig. 17:**
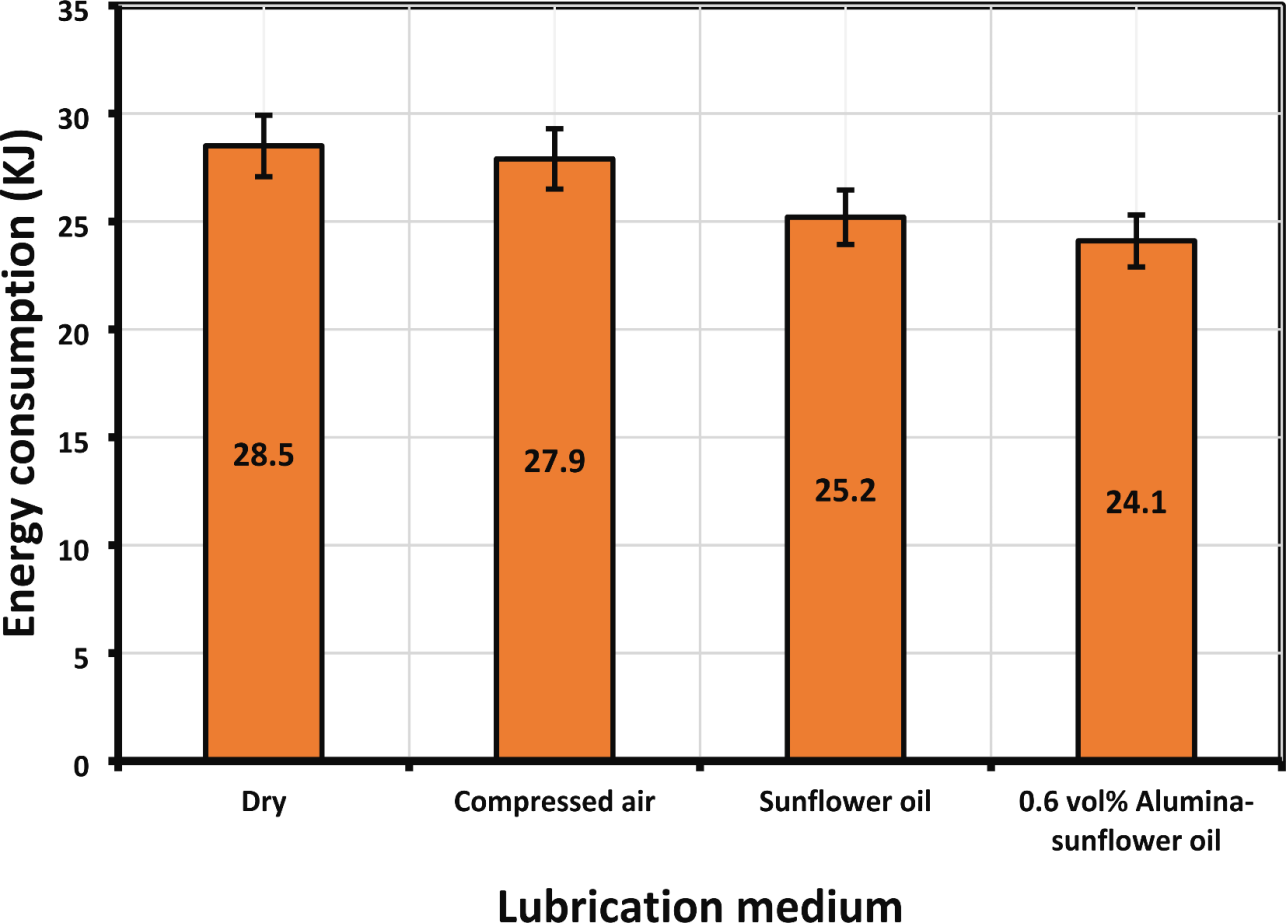
Energy consumption values in different lubricating medium.

According to one estimate by Zhao et al.^49^, industry accounts for 31% of total energy consumption, with the manufacturing sector responsible for about 60% of this energy use. They found that energy efficiency is suboptimal, as actual machining consumes only 15% of the total energy. Hence, reducing energy usage in machine tools is crucial for sustainable machining operations. In a comparative analysis of lubrication methods, the use of a 0.6 vol% Alumina-sunflower bio-oil-based medium was found to consume approximately 2.10%, 11.58%, and 15.44% less energy than dry, compressed air, and sunflower bio-oil-based machining, respectively (Fig. 17). The incorporation of nanoparticles into vegetable oil enhances its lubrication properties and thermal conductivity. This results in improved heat dissipation and reduced friction between the cutting tool and workpiece, effectively lowering the machining forces and, subsequently, the energy required for the operation. Furthermore, the enhanced lubrication properties lead to reduced wear on cutting tools, extending their lifespan and reducing the frequency of tool changes, which also contributes to energy savings.

The effects of environmental degradation have become increasingly pronounced since the onset of the Industrial Revolution. This can be attributed to the rapid expansion of production and the widespread preference for fossil fuels as the primary energy source. The extensive utilization of fossil fuels has led to a significant escalation in the concentration of greenhouse gases, particularly carbon dioxide (CO_2_), which is the primary contributor to climate change among all greenhouse gas emissions. Addressing this issue requires two key strategies: increasing the proportion of renewable energy sources in energy generation and conducting a comprehensive assessment of energy-intensive industrial processes. In essence, carbon emissions are quantified using Eq. (7):

7 $$C_{E} = E_{C} \times F_{E}$$

**Fig. 18:**
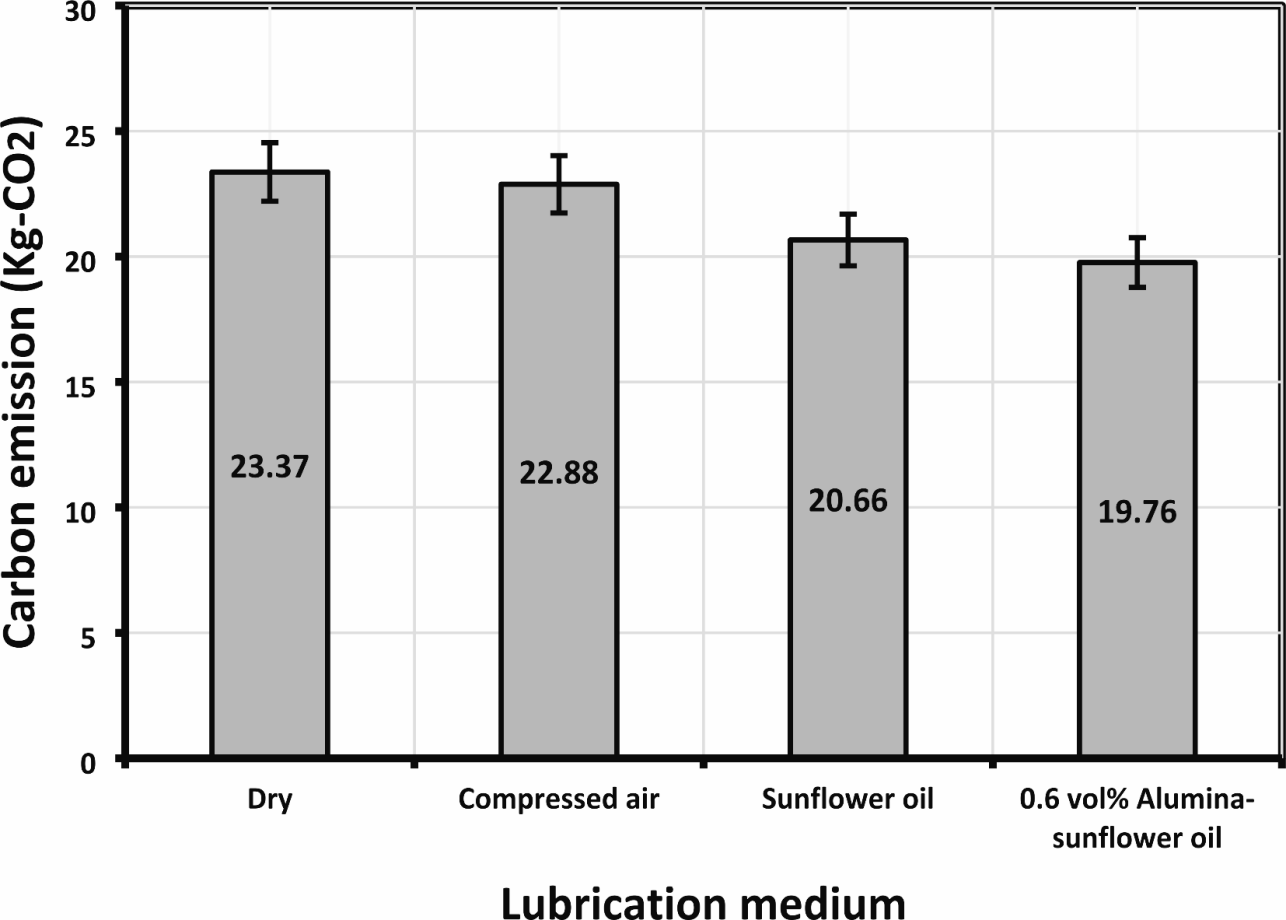
Carbon emission values in different lubricating medium.

In this study, *C*_*E*_ represents the carbon emissions measured in kilograms of CO_2_, *E*_*C*_ denotes electrical energy consumption, and *F*_*E*_ is the carbon emission factor set at 0.82 (as per the data provided by the Central Electricity Authority of India in 2021), specifically used for electric consumption. The investigation focused on assessing CO_2_ emissions generated by machine tools during machining operations, as depicted in Fig. 18. Since carbon emissions are intricately linked to energy usage, a trend analogous to the findings in the energy consumption section emerged in this analysis. The utilization of an Alumina-sunflower bio-oil-based medium has proven to be a promising strategy for diminishing carbon emissions in milling operations when compared to conventional alternatives like dry machining, compressed air, and traditional sunflower bio-oil mediums. This success can be attributed to the medium’s exceptional ability to reduce tool wear, subsequently decreasing machining time. The introduction of Alumina enhances the lubrication and cooling properties of sunflower bio-oil, leading to reduced friction and heat generation during milling. This, in turn, significantly extends the tool’s lifespan, reducing the need for frequent tool replacements and, consequently, curbing the overall machining time. Since machining time is directly associated with electrical energy consumption, the utilization of Alumina-enriched sunflower bio-oil results in reduced carbon emissions. The study revealed that a 0.6 vol% Alumina-sunflower bio-oil-based lubricating medium reduced carbon emissions by 2.10%, 11.60%, and 15.44% when compared to dry machining, compressed air, and sunflower bio-oil-based alternatives, respectively.

### Assessment of sustainable manufacturing environment

#### Description of fuzzy-Mamdani model

**Fig. 19:**
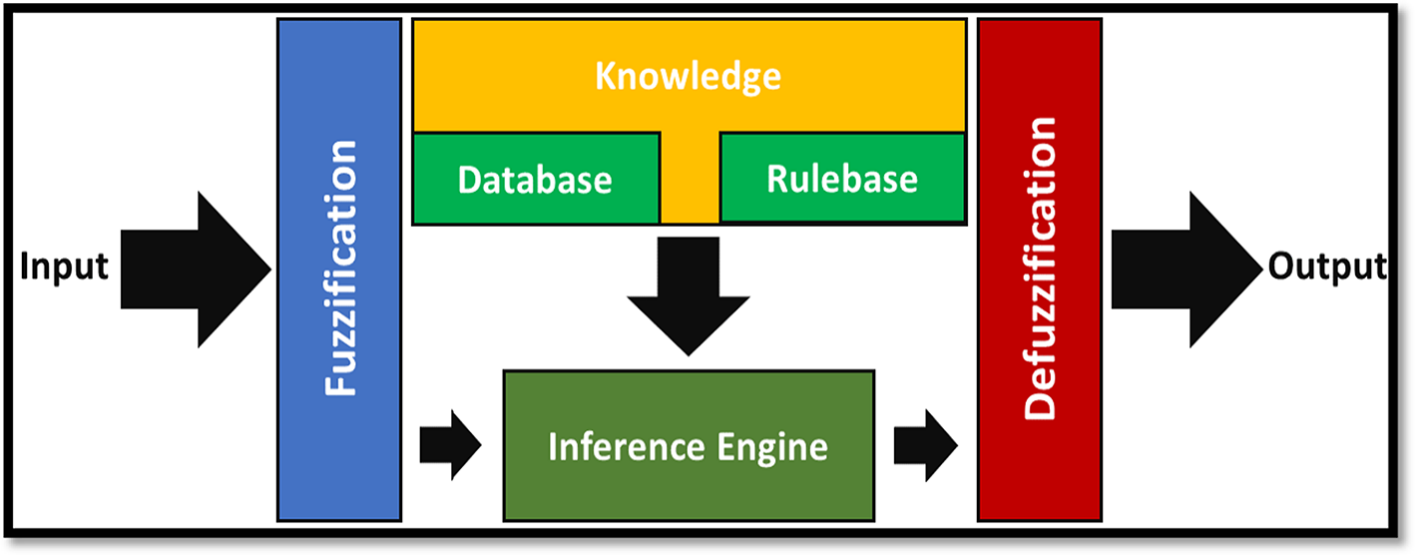
A block diagram of the applied Fuzzy-Mamdani model.

In the domain of cleaner production, the evaluation methodology for sustainability plays a crucial role in various manufacturing industries. Sustainability, in broad terms, encompasses a constructive approach that addresses current needs while confirming the capacity of future generations to fulfil their own requirements^50^. Moreover, the concept of “Sustainable or Cleaner Manufacturing” goes beyond the product itself and encompasses economic and environmental considerations. This particular study employs a Fuzzy-Mamdani model to establish a sustainable manufacturing environment for superalloy machining. The Fuzzy-Mamdani model leverages existing knowledge through fuzzy logic to derive novel insights. It involves establishing a mapping from input to output, which forms the basis for decision-making and pattern recognition^51^. The approach depends on human interpretation of data, expertise, and similar factors to generate solutions for existing problems. The proposed approach employs fuzzy inference to develop a fuzzy index that evaluates the sustainability of the machining runs. The initial phase involves defining input and output parameters in consultation with experts. Their input is invaluable for determining and fine-tuning these parameters, as well as establishing membership functions crucial to the inference model. The experts quickly reached an agreement on the linguistic values and thresholds, drawing from their collective experience. Subsequently, fuzzy variables and membership functions were described to formulate the IF-THEN fuzzy rule base. The number of fuzzy rules depends upon the possible combinations of membership functions. For a Fuzzy-Mamdani model with j-input variables, the rule count is determined by r = p^j^, where p represents the number of linguistic terms per input variable. As the system’s dimension and complexity increase, the rule base size grows exponentially^52^. The model employs a fuzzy index as the output variable, representing the sustainability level of a specific experiment. Here, the Mamdani-type inference approach is applied, calculating a weighted average to compute the precise output. The block diagram of the Fuzzy-Mamdani model implemented in this study is illustrated in Fig. 19.

#### Testing of model

In the present study, a “lower-the-better” type signal-to-noise (S/N) ratio is employed^53^, which is defined by Eq. (8), to optimize the arrangement of quality factors including surface roughness, tool wear, machining cost, and energy consumption. Subsequently, the S/N values were normalized to a range of 0 to 1 using the normalization formula specified in Eq. (9). To assess the sustainability of the machining environment, triangular fuzzy membership functions were utilized to determine the Multi-Performance Characteristics Index (MPCI). The highest MPCI value signifies the most sustainable machining environment. Each input parameter was classified into low (L), medium (M), and high (H) categories, while the output (MPCI) was segmented into very low (VL), low (L), medium (M), high (H), and very high (VH). Fuzzy IF-THEN control rules were established for the four input variables and one output variable, as depicted below:

##### Rule 1

If surface roughness is H and tool wear is H, and machining cost is H, and energy consumption is H then MPCI is VH otherwise.

##### Rule 2

If surface roughness is L and tool wear is H, and machining cost is H, and energy consumption is H then MPCI is H otherwise


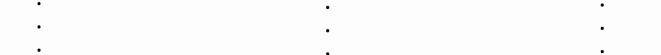


##### Rule 81

If surface roughness is M and tool wear is M, and machining cost is M, and energy consumption is M then MPCI is M otherwise.

**Fig. 20:**
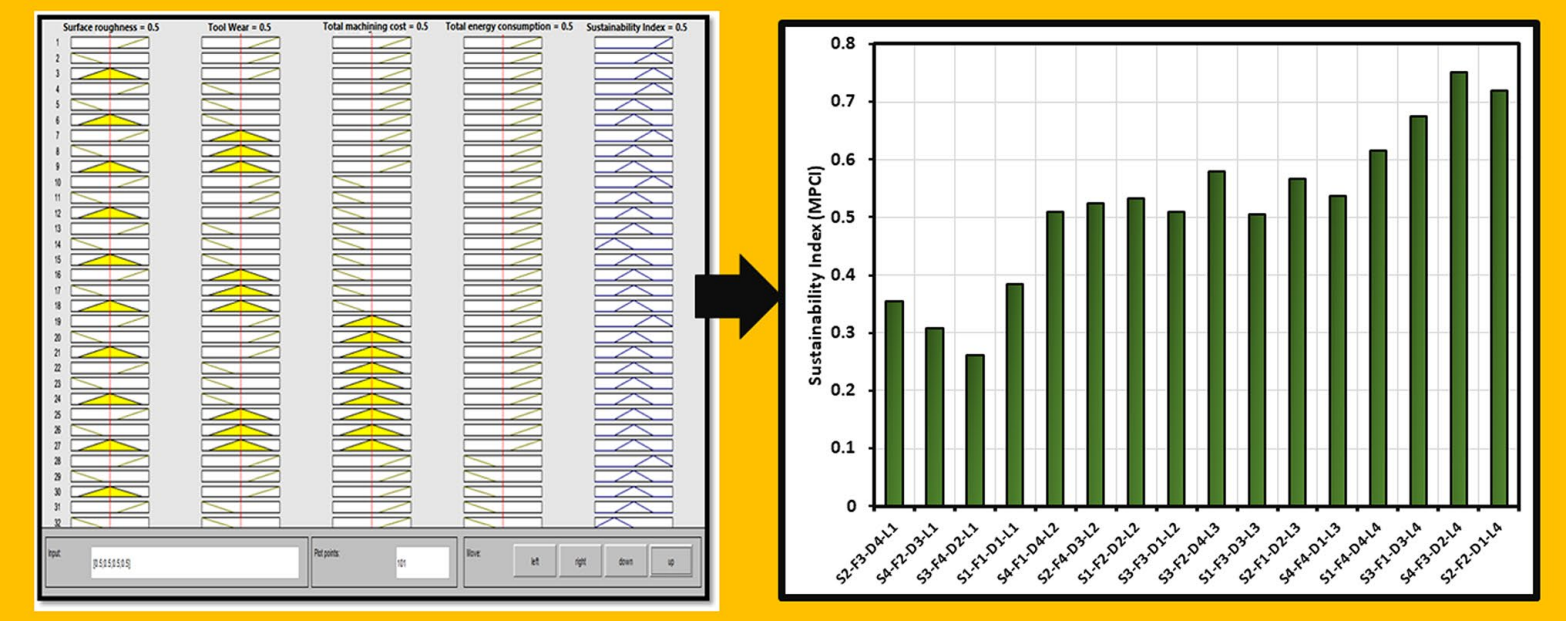
The Fuzzy-Mamdani model generated MPCI values for each experimental run.

Figure 20 displays the MPCI values generated by MATLAB for each experimental run, indicating that the highest MPCI value can be achieved by employing a 0.6 vol% Alumina-sunflower bio-oil-based lubricating medium with a speed of 75 m/min, feed rate of 0.050 mm/tooth, and depth of cut of 0.15 mm. The study demonstrates that the Fuzzy-Mamdani model can effectively contribute to sustainability management in machining operations. The employed model relies on systematic observation and verification of factors that impact the outcomes, rendering it a transparent tool that can be continuously updated with new knowledge. This characteristic sets it apart from a “black box” decision-making model, allowing for greater understanding and adaptability.

8 $$\frac{S}{N} = - 10 log\frac{{\mathop \sum \nolimits_{i = 1}^{n} {1 \mathord{\left/ {\vphantom {1 {y^{2} }}} \right. \kern-0pt} {y^{2} }}}}{n}$$

where *n* = number of experimental runs, *y* = measured value of characteristics.

9 $$X_{normalized} = \frac{{X_{i} - X_{min} }}{{X_{max} - X_{min} }}$$

where *X*_*max*_ and *X*_*min*_ signify the maximum and minimum values in the data set

## Conclusions

The present investigation has been crafted to evaluate the lubricating efficacy of an indigenous Alumina-enriched lubricant during the milling process of Hastelloy C-276. In light of the outcomes obtained through this study, the ensuing deductions can be derived:

- Increasing the vol% of reinforced Alumina improves the physical properties of sunflower bio-oil. The highest dynamic viscosity and lowest contact angle values were perceived at a 0.6 vol% nanoparticle concentration. However, beyond this concentration, no further improvement in physical behavior was observed, which may indicate the inactivity of nano-Alumina.
- Under dry conditions, the machining process ensued in the highest values of surface roughness (1.3 μm), and tool wear (0.84 mm). However, when using 0.6 vol% Alumina-sunflower bio-oil, these variables decreased by 73.31%, and 82.14%, respectively.
- SEM micrograph and EDX spectrum analysis indicated that adhesive and abrasive wear were the leading wear mechanisms in all lubricating media. Adhesion of work material on cutting inserts resulted in excessive notch wear while coating peel-off, fracture, and diffusive wear caused tool rake face wear for Hastelloy C-276. The excellent wettability and surface tension behavior of the Alumina-enriched lubricant led to less severe wear on the AlTiN-coated tool.
- Surface elemental mapping of the machined zone under 0.6 vol% Alumina-sunflower bio-oil based lubricating condition showed the development of thin surface protective films. The existence of nanoparticles improved the viscosity of sunflower bio-oil, which may have contributed to the development of these thin films.
- An exhaustive evaluation of economic and ecological factors reveals that nanoparticle-enhanced vegetable oil outperforms dry, compressed air, and sunflower bio-oil-based medium. In particular, sunflower bio-oil mixed with 0.6 vol% Alumina shows a significant reduction of 17.86% in total machining costs, along with a 15.44% decrease in both energy consumption and carbon emissions when compared to the dry medium.
- Finally, the Fuzzy-Mamdani model demonstrated that a cutting speed of 75 m/min, feed of 0.050 mm/tooth, depth of cut of 0.15 mm, and 0.6 vol% Alumina-sunflower bio-oil-based lubricating medium resulted in the most sustainable manufacturing environment, with the highest MPCI value of 0.75.

Nano-MQL has promising benefits but also presents several technical limitations. One key issue is the dispersion stability of nanofluids, as nanoparticle agglomeration over time can impact consistency, reducing the efficacy of lubrication and cooling. Additionally, the penetration performance of nanofluids in MQL is limited by the fluid’s ability to reach deep, complex cutting zones, which may hinder its effectiveness in high-precision machining applications. Moreover, the excessive oil mist concentration in Nano-MQL setups raises environmental and health concerns due to the potential release of fine particles into the air. Thus, the future research should focus on enhancing nanoparticle stability, optimizing fluid penetration to improve cooling performance, and developing environmentally friendly oil mist management techniques. These efforts could address current limitations and expand the application of MQL technology for more sustainable and efficient machining processes.

## Data Availability

The necessary data used in the manuscript are already present in the manuscript.
